# Maternal Height-standardized Prevalence of Stunting in 67 Low- and Middle-income Countries

**DOI:** 10.2188/jea.JE20200537

**Published:** 2022-07-05

**Authors:** Omar Karlsson, Rockli Kim, Barry Bogin, SV Subramanian

**Affiliations:** 1 Takemi Program in International Health, Harvard T.H. Chan School of Public Health, Harvard University, Boston MA, USA; 2 Department of Economic History, School of Economics and Management, Lund University, Lund, Sweden; 3 Division of Health Policy & Management, College of Health Science, Korea University, Seoul, South Korea; 4 Interdisciplinary Program in Precision Public Health, Department of Public Health Sciences, Graduate School of Korea University, Seoul, South Korea; 5 Harvard Center for Population and Development Studies, Cambridge MA, USA; 6 UCSD/Salk Center for Academic Research and Training in Anthropogeny, University of California, San Diego, CA, USA; 7 School of Sport, Exercise & Health Sciences, Loughborough University, Leicestershire, United Kingdom; 8 Department of Social and Behavioral Sciences, Harvard T.H. Chan School of Public Health, Harvard University, Boston MA, USA

**Keywords:** prevalence of stunting, maternal height, low and middle-income countries, undernutrition

## Abstract

**Background:**

Prevalence of stunting is frequently used as a marker of population-level child undernutrition. Parental height varies widely in low- and middle-income countries (LMIC) and is also a major determinant of stunting. While stunting is a useful measure of child health, with multiple causal components, removing the component attributable to parental height may in some cases be helpful to identify shortcoming in current environments.

**Methods:**

We estimated maternal height-standardized prevalence of stunting (SPS) in 67 LMICs and parental height-SPS in 20 LMICs and compared with crude prevalence of stunting (CPS) using data on 575,767 children under-five from 67 Demographic and Health Surveys (DHS). We supplemented the DHS with population-level measures of other child health outcomes from the World Health Organization’s (WHO) Global Health Observatory and the United Nations’ Inter-Agency Group for Child Mortality Estimation. Prevalence of stunting was defined as percentage of children with height-for-age falling below −2 z-scores from the median of the 2006 WHO growth standard.

**Results:**

The average CPS across countries was 27.8% (95% confidence interval [CI], 27.5–28.1%) and the average SPS was 23.3% (95% CI, 23.0–23.6%). The rank of countries according to SPS differed substantially from the rank according to CPS. Guatemala, Bangladesh, and Nepal had the biggest improvement in ranking according to SPS compared to CPS, while Gambia, Mali, and Senegal had the biggest decline in ranking. Guatemala had the largest difference between CPS and SPS with a CPS of 45.2 (95% CI, 43.7–46.9%) and SPS of 14.1 (95% CI, 12.6–15.8%). Senegal had the largest increase in the prevalence after standardizing maternal height, with a CPS of 28.0% (95% CI, 25.8–30.2%) and SPS of 31.6% (95% CI, 29.5–33.8%). SPS correlated better than CPS with other population-level measures of child health.

**Conclusion:**

Our study suggests that CPS is sensitive to adjustment for maternal height. Maternal height, while a strong predictor of child stunting, is not amenable to policy interventions. We showed the plausibility of SPS in capturing current exposures to undernutrition and infections in children.

## INTRODUCTION

Early-life undernutrition and repeated infections have long-term negative effects on health and socioeconomic status (SES) and are reflected in reduced physical growth during childhood and eventually shorter adult stature.^1^ Population-level undernutrition in children is commonly measured using prevalence of stunting,^2^^,^^3^ which is defined as the percentage of children with height-for-age below −2 standard deviations (SD) from the median of the World Health Organization’s (WHO) 2006 growth chart.^4^ The 2006 WHO growth chart is based on the Multicentre Growth Reference Study (MGRS) and is considered a standard of median growth that a population of healthy breastfed children ought to achieve under *optimal conditions*.^5^ Prevalence of stunting is most commonly interpreted as nutrition deficits from inadequate diet and repeated infections.^2^^,^^3^ Inadequate psychosocial stimulation is also suggested to cause stunting.^6^ However, paternal and especially maternal heights are the strongest and most consistent observable predictors of child stunting.^7^^–^^9^

Harmful exposures during parents’ early-life, reflected in their adult height, can negatively affect child growth via various pathways, such as maternal health, biomechanical and biological mechanisms (eg, intrauterine growth restrictions and prematurity), as well as SES^1^^,^^11^^,^^12^ and other social-economic-political-emotional (SEPE) factors.^13^^,^^14^ The intergenerational effects of harmful exposures on child health may even stretch beyond two generations.^10^ Genetic (including epigenetic) and hormonal factors may also link parental height and offspring growth.^15^^–^^17^

Regardless of exact links, parental height is a non-modifiable determinant of child stunting. Therefore, the prevalence of stunting reflects not only current environmental conditions—such as nutrition and infections—but also parental height.

Because adult height varies across countries, the extent to which the prevalence of stunting reflects parental stature likely varies as well. From a policy perspective, it is helpful to distinguish between the prevalence of child stunting attributable to the current environment on the one hand, and parental height on the other. With this motivation, we provide maternal height-standardized prevalence of stunting (SPS) in 67 low- and middle-income countries (LMICs). Maternal height is more widely available than paternal height, but we also provide parental height-SPS for 20 LMICs. We compared the rank correlations of SPS and crude prevalence of stunting (CPS) with other population-level measures of child health. Bringing this precision into evaluation and deliberation is helpful for developing the appropriate policy instruments to fight stunting.

## METHODS

### Data source

The data for the analyses came from the Demographic and Health Surveys (DHS) which are nationally representative household surveys conducted in LMICs.^18^ The surveys generally used a stratified two-stage sampling design from a sampling frame based on the most recent census. Primary sampling units (PSU) consisted of villages in rural areas and census enumeration blocks in urban areas. PSU were selected with a probability proportional to size from strata of sub-national geographic or administrative regions separated into urban and rural areas. In the second stage, around 20–30 households from each PSU were sampled and women aged 15–49 were interviewed.^19^ Response rates typically exceed 90%.^18^^,^^20^

The heights of interviewed women and their children under 5 years old were measured (sometimes only in a sub-sample). Heights of a men aged 15–54 years old were measured in 20 countries (in a sub-sample). We included the most recent survey that included maternal and child heights for each country. Survey years ranged from 1994 to 2018, with 53 conducted after 2010 and 61 after 2005 (eTable 1). The full sample consisted of 646,417 children, while the final sample used for analysis consisted of 575,767 children (or 89% of the full sample). For children, 91.5% had a valid height measure, while 95.9% of mothers had a valid height measure (eTable 2). Children excluded from the analysis due to missing or implausible height measures appear to show fairly small differences in observable characteristics (eTable 3).

In addition, we supplemented the DHS data with country-level measures of child health and health care access from WHO’s Global Health Observatory (GHO): under-5 year old death rates (ie, deaths per 1,000 live births) from acute lower respiratory infections (ALRI) and diarrhea, available annually for 2000–2017; and prevalence of anemia (%) among children under 5 years old and diphtheria-tetanus-pertussis immunizations (DPT3) coverage (%) among 1-year-olds, available for 1990–2017.^21^ We also used under-5 mortality rate (U5MR; ie, deaths of children under 5 years of age per 1,000 live births) and child mortality rate (CMR; ie, deaths of children 1–4 years old per 1,000) from United Nations’ (UN) Inter-Agency Group for Child Mortality Estimation (IGME).^22^ ALRI and diarrhea are major health problems and have been estimated to cause 47% and 35% of disability adjusted life years (DALY) from growth failure in children,^23^ respectively, and are major causes of under-5 deaths.^24^ Although some of these measures, at the individual level, may have an association with maternal height (eg, U5MR), it appears to be lower than for stunting.^8^^,^^9^

### Outcome

Stunting was defined as height-for-age z-scores (HAZ) below −2 SD from the WHO 2006 reference median.^4^ Height was measured in centimeters with one decimal and age was measured in months. We excluded children with implausible values—HAZ above 6 SD or below −6 SD.^25^

At the individual level, HAZ −2 SD is not a meaningful cut-off point for growth faltering or associated risk factors (eg, mortality^26^ and cognitive and motor development^27^): instead, prevalence of stunting is a summary measure of population level growth deficits, since only ∼2.3% are expected to have HAZ below −2 SD, and any excess indicates shortcomings, relative to children in the MGRS.

### Standardization variables

Heights of adult females and, in some surveys, males were measured in centimeters with one decimal. We round parental height to the nearest centimeter to reduce the number of strata used for standardization. We excluded parents with implausible height—HAZ above 6 SD or below −6 SD from a normative median height.^28^ We used the distribution (mean and standard deviation, assuming a normal distribution^29^^–^^31^) of parental height in the MGRS sample as a reference for standardization. In the MGRS (cross-sectional sample), females were 161.0 cm (SD, 7.2 cm) and males were 173.8 cm (SD, 7.9 cm) tall on average.^5^

### Estimation

CPS was estimated as the percentage of children falling below −2 SD (z-scores) from the WHO growth reference. We then standardized stunting prevalence according to maternal height in the reference population (ie, the MGRS). Maternal height-SPS is defined as:

$$\text{SPS}=\sum_{\text{cm}=\text{min}}^{\text{max}}{\text{cps}}_{\text{cm},t}\times {\text{pd}}_{\text{cm},r}$$ (1)

where *cps_cm_*_,_*_t_* is the crude prevalence of stunting at each *cm* of maternal height in the target sample and *pd_cm_*_,_*_r_* is the probability-density for each *cm* of maternal height in the reference population. When standardizing maternal height only, the *pd_cm_*_,_*_r_* was obtained using the mean and standard deviation of mothers in the MGRS.

We then compared the Spearman’s rank correlation coefficients for CPS and maternal height-SPS with other country-level measures of child health: diarrhea mortality rate, ALRI mortality rate, anemia, U5MR, CMR, and DPT3, obtained from the GHO and IGME. No causality was attributed to these correlations. We limited extrapolation of the GHO data to 2 years before and after the last year with available data. We linked the country level measures to the year of each DHS survey: averaged over year of survey and the 4 years preceding the survey to reduce noise and to match the exposure period of children in the DHS data.

All estimates were weighted using DHS sampling weights and 95% confidence intervals (CIs) were calculated using robust standard errors adjusted for clustering at the PSU-level for CPS and PSU-level crossed with maternal height strata for SPS, using clustered sandwich estimator of variance. We used logit transformation to ensure that CIs were between 0 and 100% for the prevalence of stunting. The sampling weights were scaled to add up to one for each survey, so each survey contributes equally to the pooled estimates regardless of sample size and population. For maternal-height SPS, sampling weights were re-scaled to sum up to the probability-density within each stratum of maternal height in the reference population. When discussing results, we used the term standardize to mean imposing a height distribution from the MGRS reference population and adjustment (discussed in our sensitivity analyses) to mean holding our measure of living standards (household wealth) at its sample mean.

We do eight sensitivity analyses (see eAppendix 1), where we: 1) account for the correlation between living standards and maternal height (eFigure 1); 2) predict prevalence of stunting at the MGRS mean from logit models of stunting on maternal height to avoid potential problems with direct standardization (eFigure 2); 3) exclude outliers in terms of maternal height (eFigure 3 and eFigure 4); 4) estimate maternal height-standardized mean child HAZ (eFigure 5); 5) impute missing information on stunting using multiple imputations chained equations (eFigure 6); 6) exclude children born to mothers under 25 years old at the time of birth (eFigure 7); 7) use child wasting as a placebo outcome, showing maternal height-standardized prevalence of wasting compared to crude prevalence of wasting (eFigure 8); and 8) show Pearson’s correlation coefficients instead of Spearman’s rank correlation coefficients, for the relationship of SPS and CPS with other country-level measures of child health (eFigure 9).

Additionally, we show results from supplementary analyses (see eAppendix 2). We show the association between maternal height and child stunting from regression models in each country; both using original sampling weights for crude prevalence, as well as sampling weights re-scaled according to the distribution of maternal height in the reference population for standardized prevalence (eTable 4). We also present results where we standardized according to height of both parents (eTable 5, eTable 6, and eTable 7 for information on the male sub-sample, and eFigure 10, eFigure 11, and eTable 8 for results).

## RESULTS

### Maternal height distribution

**Figure 1.**
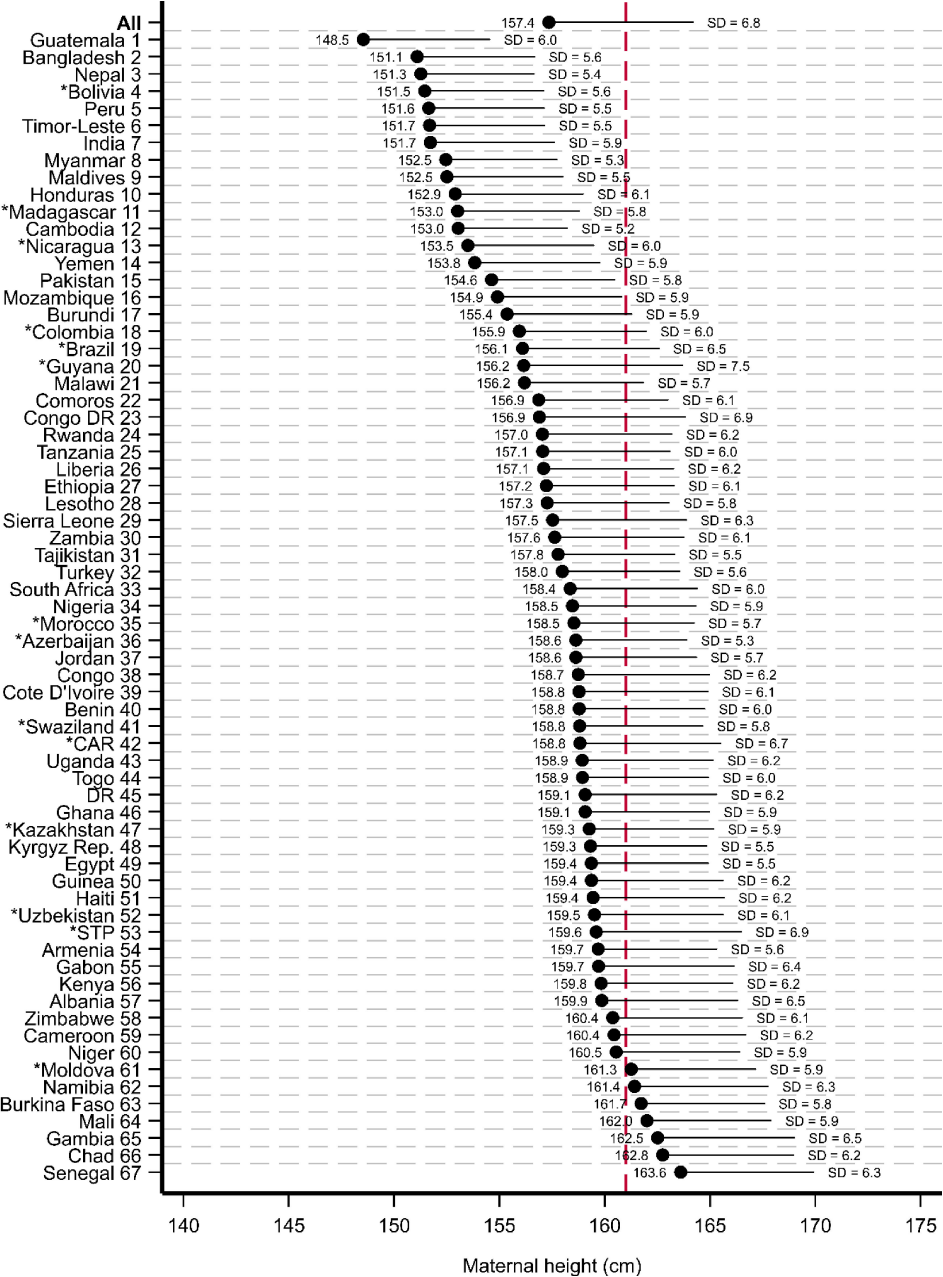
Average maternal height and standard deviations Notes: See eTable 9 for tabulated estimates with 95% confidence intervals. ^*^Indicates estimates from surveys conducted before 2010. Black dots show means. Black lines extending from means indicate one standard deviation (SD). Red dashed line shows MGRS mean. All estimates were weighted using sampling weights which sum up to 1 for each survey. CAR, Central African Republic; Congo DR, Democratic Republic of Congo; STP, Sao Tome and Principe; DR, Dominican Republic; Rep, Republic.

Overall, mothers in our sample were 157.4 cm (SD, 6.8 cm) on average (Figure 1 and eTable 9). The shortest mothers were in Guatemala (148.5 cm; SD, 6.0 cm) while the tallest mothers were in West-Africa, especially Senegal (163.6 cm; SD, 6.3 cm). Mothers in our analytical sample were shorter than mothers in the MGRS in all but seven countries, six of which are in sub-Saharan Africa.

### Maternal height-standardized prevalence of stunting

**Figure 2.**
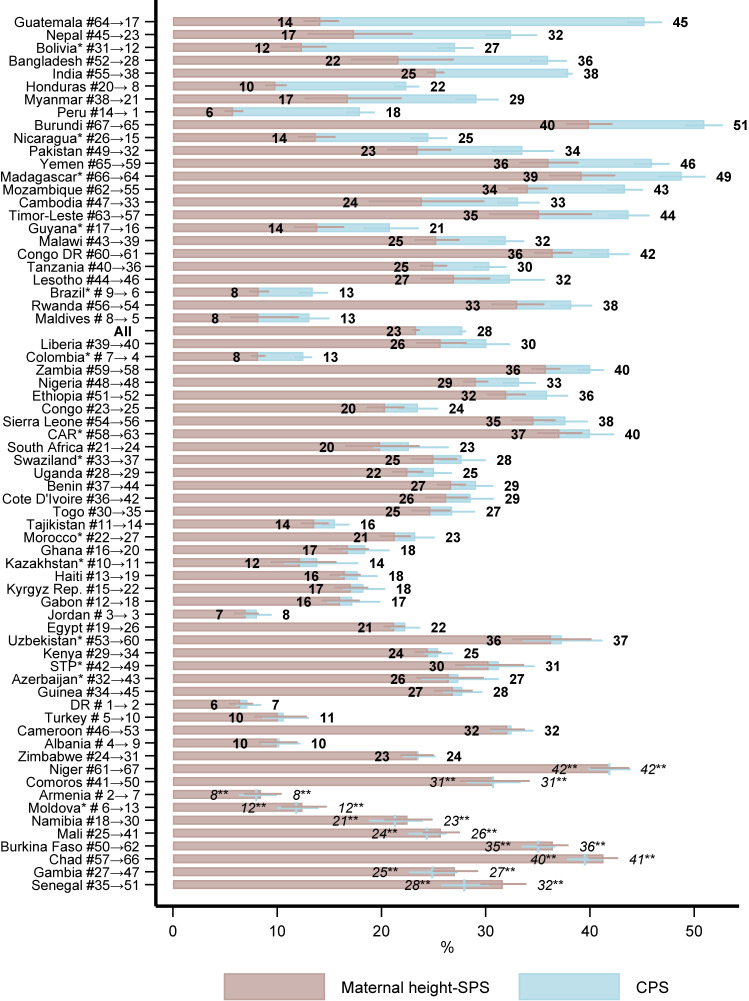
Crude and maternal height-standardized prevalence of stunting Notes: See eTable 10 for tabulated estimates and rankings. Countries are ordered (from large to small) according to the difference between crude prevalence of stunting (CPS) and maternal height-standardized prevalence of stunting (SPS). # indicates rank (from low to high) according to CPS and → indicates rank according to SPS. ^*^Indicates estimates from surveys conducted before 2010. ^**^ Indicates countries where CPS was higher than SPS. All estimates were weighted using sampling weights which sum up to 1 for each survey. For SPS, sampling weights were re-scaled to sum up to the probability-density within each stratum (cm) of maternal height in the reference population. Standard errors were adjusted for clustering at the PSU-level when estimating CPS and PSU crossed with maternal height when estimating SPS. 95% confidence intervals are shown. CAR, Central African Republic; Congo DR, Democratic Republic of Congo; STP, Sao Tome and Principe; DR, Dominican Republic; Rep, Republic.

The CPS was 27.8% (95% CI, 27.5–28.1%) in the pooled sample (Figure 2 and eTable 10). The CPS was the greatest in Burundi, or 51% (95% CI, 49.3–52.7%), and the lowest in the Dominican Republic (DR), 7.1% (95% CI, 6.1–8.4%). After standardizing maternal height, the SPS was 23.3% (95% CI, 23.0–23.6%) in the pooled sample.

Burundi also had one of the highest SPS (39.9%; 95% CI, 37.8–42.1%) but other sub-Saharan African countries, Niger (41.9%; 95% CI, 40.1–43.7%) and Chad (41.3%; 95% CI, 40.0–42.6%) had a greater prevalence. DR also had one of the lowest SPS (6.4%; 95% CI, 5.4–7.6%), but Peru had slightly lower SPS (5.8%; 95% CI, 5.0–6.7%).

The biggest deterioration in the ranking of countries according to prevalence of stunting after standardizing maternal height was for West-Africa, especially Mali, Gambia, and Senegal, where the ranking deteriorated by between 16 to 20 places. The Latin American country of Guatemala had the biggest gains in terms of ranking according to prevalence of stunting after standardizing and improved by 47 places. The South Asian countries, especially Nepal, Bangladesh, and India, but also Pakistan, showed large improvements in the ranking, by between 17 to 24 places. Overall, European and African countries generally decreased in the ranking, while Asian and Latin American countries improved.

### Comparing the correlation of CPS and SPS with other measures of child health

**Figure 3.**
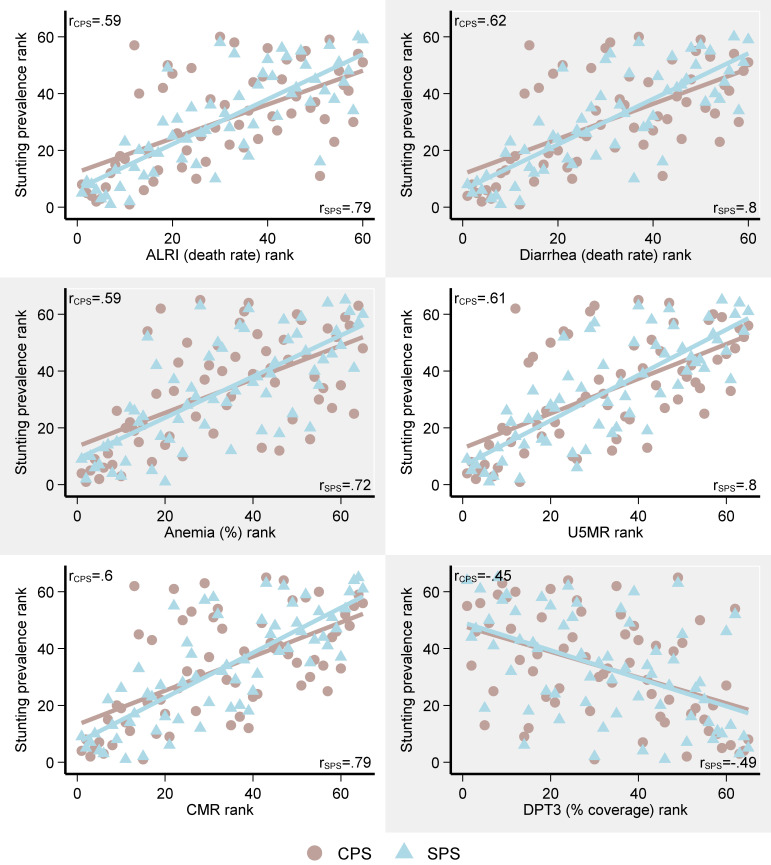
Scatterplots and Spearman’s rank correlation coefficients for ranked CPS and maternal height-SPS and other ranked aggregate measures of child health Notes: See eTable 11 for tabulated estimates. Spearman’s rank correlation coefficients are shown. Acute lower respiratory infections death rate for children under five (ALRI); Diarrhea death rate for children under 5 (Diarrhea); Prevalence of anemia among children under 5 years old (Anemia); Under-5 mortality rate (U5MR); Child mortality rate (CMR); Diphtheria-pertussis-tetanus vaccination coverage for children 1–2 years old (DPT3).

The maternal height-SPS rank correlated better than CPS with ALRI (rho 0.79 vs 0.59), diarrhea (*r* = 0.80 vs 0.62), anemia (*r* = 0.72 vs 0.59), U5MR (*r* = 0.8 vs 0.61), and CMR (*r* = 0.79 vs 0.6), while the difference was small for DPT3 (*r* = −0.49 vs −0.45) (Figure 3 and eTable 11).

### Sensitivity analyses

There were some noteworthy results from our sensitivity analyses. Adjusting for the correlation between maternal height and household living standards changed the SPS in some cases: the adjusted estimates ranged from being four percentage points greater to five percentage points smaller than unadjusted, with an average absolute difference of less than 1 percentage point (eFigure 1).

Predicted stunting, from logit models of stunting on maternal height, shows small differences compared to SPS (eFigure 2): predicted stunting appears to be generally lower than SPS, ranging from being 5 percentage points lower to 1 percentage point greater, with an average absolute difference of 1.4 percentage points.

### Supplementary analyses

There were some noteworthy results from our supplementary analyses. In the sub-sample for males, the average male height was 168.9 cm (SD, 7.5 cm), ranging from 161.7 cm (SD, 5.9 cm) in Timor-Leste to 174.96 cm (SD, 7.0 cm) in Senegal (eTable 7). Parental height-SPS was two percentage points lower than maternal height-SPS in the pooled sample and ranged from being 12 percentage points lower in Timor-Leste to 0.3 percentage point greater in Senegal (eFigure 10).

## DISCUSSION

Our analysis presents three salient findings. First, over the 67 countries in our sample, we found an equally-weighted average CPS of 28%, which was reduced to 23% after standardizing maternal height using the MGRS as a reference population. Second, we found substantial reranking of countries according to residual stunting prevalence after standardizing maternal height. In some sub-Saharan African countries, tall average maternal height suppresses the prevalence of stunting, potentially masking the full extent of the harmfulness of the current environment children are exposed to. By contrast, in India, for example, a large share of the prevalence of stunting reflected short maternal height, although the SPS still indicates severe shortcomings in the current environment. Finally, we found that the maternal height-SPS correlated better than CPS with other country-level measures of child health, specifically diarrhea and ALRI mortality rates, anemia, U5MR, and CMR, demonstrating the plausibility of SPS capturing current exposures to undernutrition and infections.

Lack of information on mother’s living standards in early life was a limitation of this study. Ideally, we would have also tested the sensitivity of our results to adjustments for childhood living standards of mothers, in addition to adult living standards, since adult and childhood living standards are correlated.^32^ While we wanted to standardize the prevalence of stunting according to maternal height and adjust for the component of maternal height reflecting current living standards in our sensitivity analysis, we did not want our living standards adjustment to remove any early-life determinants of maternal height. Therefore, our living standards adjustments may overcorrect our standardized measures. We do, however, find that adjusting for living standards had only a small impact on the SPS overall. Further, research has found measures of adult SES to explain less than 2% of the variation in adult height in most countries.^33^ It should be kept in mind that child stunting and maternal height may share risk factors from correlated environments^32^ beyond what is captured by our measure of current living standards.

India has persistently high stunting prevalence,^34^ which is even higher than in most countries in sub-Saharan Africa that perform far worse on other measures, such as U5MR.^35^ Tall stature of parents in sub-Saharan Africa^36^ could explain why India has similar or greater CPS than most sub-Saharan African countries, for example, Sierra Leone (38% both in Sierra Leone and India), while doing much better on other indicators, such as U5MR (137 per 1,000 live births in Sierra Leone vs 44 in India).^37^ Mothers in India were shorter than mothers in Sierra Leone (157.5 cm in Sierra Leone vs 151.7 cm in India), and after standardizing maternal height, India had an SPS of 25% while Sierra Leone had an SPS of 35%. Results were similar when comparing other South Asian countries to other countries in sub-Saharan Africa.

We do not undermine the importance of the measure of child stunting prevalence as an indicator of child health. Further, what we refer to as *crude* prevalence of stunting in the paper, does indeed measure prevalence of stunted growth. SPS, in its calculation, simply reduces the weight on children born to mothers of a stature that is less common in the reference (which is the MGRS in our study) than in the target population. Since most populations are shorter than the MGRS, and children born to shorter mothers are more likely to be stunted, in effect, the weight on stunted children is usually reduced in the calculation of SPS. Further, we are not *correcting* the measure for stunting prevalence, and SPS does not capture the *real* prevalence of stunting better (worse in fact) than the crude measure: It is a different measure which may be more useful in many circumstances since it is more specific to the nutritional intake and infections children have been directly exposed to in their lifetime. However, CPS is an informative and crucial measure as it also shows the long arm of deprivation and achieving child growth close to the MGRS should remain the goal. Further, children that are small due to short maternal height can be targeted for intervention to improve their growth.

### Conclusion

Concerted efforts are needed to attain the Sustainable Development Goal of eliminating hunger by 2030.^3^ Most LMICs are still far from the stunting prevalence observed for children in ideal environments, regardless of whether we count all stunted children or reduce the weight on children with maternal height further from the MGRS average. When using stunting prevalence for evaluating the extent of modifiable risk factors causing stunted growth, removing the influence of the strongest observable determinant, maternal height, may give a better quantification of the extent of other risk factors. Maternal height is routinely collected and can be accounted for when using the prevalence of stunting to evaluate current exposures.

## SUPPLEMENTARY MATERIAL

eTable 1. Samples, survey years, and missing observations

eTable 2. Result of height measurements for pooled sample

eTable 3. Comparing attributes of children with missing and non-missing data using linear regressions

eAppendix 1. Sensitivity analyses

eFigure 1. Adjusted maternal height-standardized prevalence of stunting

eFigure 2. Prevalence of stunting predicted at the MGRS mean maternal height

eFigure 3. Linear fit and LOWESS fit of stunting on maternal height and a histogram of maternal height

eFigure 4. Maternal height-standardized prevalence of stunting: excluding maternal height outliers

eFigure 5. Maternal height-standardized mean child height-for-age z-score

eFigure 6. Maternal height-standardized prevalence of stunting: imputing missing stunting information

eFigure 7. Maternal height-standardized prevalence of stunting: excluding children born to mothers younger than 25 years

eFigure 8. Crude and maternal height-standardized prevalence of wasting

eFigure 9. Scatterplots and Pearson’s correlation coefficients for CPS and maternal height-SPS and other aggregate measures of child health

eAppendix 2. Supplementary analyses

eTable 4. Results from regression of stunting on maternal height

eTable 5. Samples, survey years, and missing observations: male sub-sample

eTable 6. Result of father’s height measurements

eTable 7. Average maternal and paternal height: male sub-sample

eFigure 10. Parental height-standardized prevalence of stunting: male sub-sample

eFigure 11. Mid-parental height-standardized prevalence of stunting: male sub-sample

eTable 8. Results from OLS regressions estimating crude, standardized, and standardized and adjusted prevalence of stunting as well as regression parameters: male sub-sample

eTable 9. Average maternal height (cm) from Figure 1

eTable 10. CPS and maternal height-SPS estimates, ranks, and change in rank from Figure 2

eTable 11. Spearman’s rank correlation coefficients of CPS and SPS and other aggregate measures of child health from Figure 3
