# Supplementary material for: Maternal Height-standardized Prevalence of Stunting in 67 Low- and Middle-income Countries
Source: J Epidemiol. 2022 Jul 5;32(7):337–44. doi: 10.2188/jea.JE20200537 (PMC9189321; doi:10.2188/jea.JE20200537)
Supplement: Supplementary file 1 [file je-32-337-s001.pdf]

Karlsson O, Rockli K, Bogin B, & Subramanian S V. **Maternal Height-Standardized Prevalence of Stunting in 67 Low- and- Middle-Income Countries**. *Journal of Epidemiology* 2021.

Supplementary Information

eTable 1. Samples, survey years, and missing observations 2

eTable 2. Result of height measurements for pooled sample 5

eTable 3. Comparing attributes of children with missing and non-missing data using linear regressions 6

eAppendix 1. Sensitivity analyses 7

eFigure 1. Adjusted maternal height-standardized prevalence of stunting 8

eFigure 2. Prevalence of stunting predicted at the MGRS mean maternal height 9

eFigure 3. Linear fit and LOWESS fit of stunting on maternal height and a histogram of maternal height 10

eFigure 4. Maternal height-standardized prevalence of stunting: excluding maternal height outliers 11

eFigure 5. Maternal height-standardized mean child height-for-age z-score 12

eFigure 6 Maternal height-standardized prevalence of stunting: imputing missing stunting information 13

eFigure 7. Maternal height-standardized prevalence of stunting: excluding children born to mothers younger than 25 years ... 14

eFigure 8. Crude and maternal height-standardized prevalence of wasting 15

eFigure 9. Scatterplots and Pearson's correlation coefficients for CPS and maternal height-SPS and other aggregate measures of child health 16

eAppendix 2. Supplementary analyses 17

eTable 4. Results from regression of stunting on maternal height 18

eTable 5. Samples, survey years, and missing observations: male sub-sample 28

eTable 6. Result of father's height measurements 29

eTable 7. Average maternal and paternal height: male sub-sample 30

eFigure 10. Parental height-standardized prevalence of stunting: male sub-sample 31

eFigure 11. Mid-parental height-standardized prevalence of stunting: male sub-sample 32

eTable 8. Results from OLS regressions estimating crude, standardized, and standardized and adjusted prevalence of stunting as well as regression parameters: male sub-sample 33

eTable 9. Average maternal height (cm) from Figure 1 44

eTable 10. CPS and maternal height-SPS estimates, ranks, and change in rank from Figure 2 47

eTable 11. Spearman's rank correlation coefficients of CPS and SPS and other aggregate measures of child health from Figure 3 49

eTable 1. Samples, survey years, and missing observations

	Year	Missing		Full sample	Final sample [%]
		Stunting	Maternal height		
All	2012 (mean)	54,521	26,595	646,417	575,767 [89.1]
Albania	2017–2018	265	36	2,755	2,475 [89.8]
Armenia	2015–2016	128	51	1,710	1,576 [92.2]
Azerbaijan	2006	183	57	2,196	2,000 [91.1]
Bangladesh	2014	536	62	7,567	7,009 [92.6]
Benin	2017–2018	275	71	6,596	6,298 [95.5]
Bolivia	2008	616	131	8,394	7,734 [92.1]
Brazil	1996	672	348	4,818	4,048 [84.0]
Burkina Faso	2010	333	59	6,984	6,630 [94.9]
Burundi	2016–2017	147	27	6,199	6,040 [97.4]
Cambodia	2014	221	24	4,592	4,361 [95.0]
Cameroon	2011	1,011	532	6,128	5,094 [83.1]
Central African Republic	1994–1995	166	44	2,561	2,369 [92.5]
Chad	2014–2015	2,281	751	12,489	10,127 [81.1]
Colombia	2009–2010	1,411	1,142	17,442	15,412 [88.4]
Comoros	2012	470	60	3,022	2,519 [83.4]
Congo	2011–2012	341	76	4,845	4,472 [92.3]
Congo Democratic Republic	2013–2014	699	163	8,902	8,114 [91.1]
Cote D'Ivoire	2011–2012	466	189	3,709	3,185 [85.9]
Dominican Republic	2013	381	56	3,606	3,205 [88.9]
Egypt	2014	1,052	113	15,641	14,542 [93.0]
Ethiopia	2016	1,067	379	10,006	8,840 [88.3]
Gabon	2012	549	53	3,965	3,392 [85.5]
Gambia	2013	608	177	3,814	3,145 [82.5]
Ghana	2014	147	21	2,883	2,727 [94.6]
Guatemala	2014–2015	300	938	12,071	10,908 [90.4]
Guinea	2018	283	57	3,766	3,455 [91.7]
Guyana	2009	465	84	2,105	1,620 [77.0]
Haiti	2016–2017	497	2,036	6,120	3,752 [61.3]
Honduras	2011–2012	591	111	10,592	9,974 [94.2]
India	2015–2016	15,033	3,283	247,743	231,829 [93.6]
Jordan	2012	359	167	6,668	6,297 [94.4]
Kazakhstan	1999	687	629	1,266	578 [45.7]

Kenya	2014	874	10,124	19,699	9,032 [45.9]
Kyrgyz Republic	2012	193	31	4,247	4,043 [95.2]
Lesotho	2014	184	14	1,516	1,330 [87.7]
Liberia	2013	417	28	3,630	3,196 [88.0]
Madagascar	2008–2009	942	392	6,191	5,220 [84.3]
Malawi	2015–2016	452	105	5,614	5,140 [91.6]
Maldives	2016–2017	680	247	3,055	2,357 [77.2]
Mali	2018	345	74	4,717	4,347 [92.2]
Moldova	2005	189	24	1,533	1,343 [87.6]
Morocco	2003–2004	343	22	5,916	5,560 [94.0]
Mozambique	2011	778	88	10,291	9,484 [92.2]
Myanmar	2015–2016	382	58	4,597	4,202 [91.4]
Namibia	2013	589	117	2,428	1,794 [73.9]
Nepal	2016	89	13	2,461	2,366 [96.1]
Nicaragua	2001	713	221	6,727	5,927 [88.1]
Niger	2012	780	265	5,728	4,872 [85.1]
Nigeria	2018	896	249	12,282	11,298 [92.0]
Pakistan	2017–2018	606	116	4,757	4,127 [86.8]
Peru	2012	221	47	9,445	9,208 [97.5]
Rwanda	2014–2015	350	174	3,915	3,559 [90.9]
Sao Tome & Principe	2008–2009	257	191	1,851	1,469 [79.4]
Senegal	2010–2011	723	293	4,530	3,738 [82.5]
Sierra Leone	2013	1,045	146	5,368	4,266 [79.5]
South Africa	2016	587	317	1,701	1,053 [61.9]
Swaziland	2006–2007	470	67	2,537	2,047 [80.7]
Tajikistan	2017	140	39	6,019	5,871 [97.5]
Tanzania	2015–2016	710	61	9,713	8,984 [92.5]
Timor-Leste	2016	1,027	77	6,956	5,894 [84.7]
Togo	2013–2014	149	30	3,361	3,202 [95.3]
Turkey	2013	2,247	364	3,592	1,334 [37.1]
Uganda	2016	501	62	4,931	4,413 [89.5]
Uzbekistan	1996	235	14	1,261	1,022 [81.0]
Yemen	2013	1,886	313	15,834	13,837 [87.4]
Zambia	2013–2014	1,442	128	13,052	11,570 [88.6]
Zimbabwe	2015	839	157	5,807	4,935 [85.0]

Notes: Percentages of full samples are shown in square brackets.

eTable 2. Result of height measurements for pooled sample

	Child height	Mother's height
Measured	591,896 (91.5%)	619,972 (95.9%)
Implausible values	10,517 (1.6%)	1,146 (0.2%)
No measure in HH	14,918 (2.3%)	13,733 (2.1%)
Mother refused	168 (0.0%)	
Not present	4,344 (0.7%)	1,424 (0.2%)
Refused	6,800 (1.1%)	4,483 (0.7%)
Sick	73 (0.0%)	
Unknown reason	17,860 (2.8%)	5,818 (0.9%)
Total	646,576	646,576

Notes: Height-for-age z-scores (HAZ) above 6 or below -6 were defined as implausible. No measure found in household (HH) usually occurs when households are not sampled for anthropometric measures but in these cases, it was not possible to establish with full certainty. Children in households not selected for height measurement were excluded.

eTable 3. Comparing attributes of children with missing and non-missing data using linear regressions

		Not missing (constant)		Missing (coefficient)	
Outcome variable	Observations	Mean	95% CI	Difference in Mean	95% CI
Maternal height, cm	619,822	157.30	157.25 to 157.34	0.13	0.01 to 0.25
Household wealth, z-score	646,417	-0.15	-0.17, -0.14	0.14	0.12 to 0.16
Female	646,417	0.49	0.49 to 0.50	-0.01	-0.02, -0.00
Age, months	646,417	28.45	28.37 to 28.52	1.81	1.54 to 2.09
Birth order	646,417	3.07	3.06 to 3.08	-0.25	-0.28, -0.22

CI, confidence interval.

Notes: Estimates were obtained from an OLS regression of the respective test variables on a binary indicator for missingness (i.e., whether a child had missing height or missing maternal height). Survey specific variation was accounted for by subtracting a survey specific mean from the indicator for missingness. The mean (i.e., the constant) shown in the table was then estimated as the predicted test variable at mean missingness. A household wealth index was provided in the DHS, calculated from a principal component analysis using ownership of various assets and amenities. We converted the resulting factor scores into survey-specific z-scores. Estimates were weighted using sampling weights scaled to sum up to one for the analytical sample in each survey. Standard errors were adjusted for clustering at the PSU-level.

eAppendix 1. Sensitivity analyses

Adults from better-off households may have received better nutrition as well as other investments (e.g., education), which may have contributed to their adult living standards as well as height. Our goal was to remove the influence of parental height and estimate prevalence of stunting which better reflects children's current exposures. However, since taller adults have higher living-standards, standardizing stunting prevalence according to parental height may remove some of the current exposures related to living-standards. Therefore, we show estimates adjusted for a household wealth index provided in the DHS, calculated from a principal component analysis using ownership of various assets and amenities (1). We converted the resulting factor scores into survey-specific z-scores. We estimated the adjusted SPS as:

$$y = \alpha + \beta_1 (m - r) + \rho(w - \overline{w}) + \varepsilon$$

adding household wealth (w) centered around the mean in the analysis sample ($\overline{w}$), in addition to maternal height centered around mean maternal height in the reference, with sampling weights re-scaled to sum up to the probability-density within each strata of maternal height in the reference population. Then $\alpha \times 100$ gives the SPS while holding household wealth at its sample mean. We show these results in **eFigure 1** in this Supplement.

We show predicted prevalence of stunting at the mean MGRS maternal height, obtained from a logit model of stunting on maternal height **(eFigure 2** in this Supplement). Direct standardization using small strata may be problematic when average maternal height in the target population is far from the average in the MGRS, and when sample sizes are small, since the standardized estimate will rely heavily on relatively few observations at the extreme end of the distribution in the target population.

The relationship between maternal height appears to be linear across most of the height distribution, except for outliers above the ~99th percentile of maternal height (**eFigure 3** in this Supplement). Linearity is, however, not a requirement for the SPS but outliers may skew the results, and this non-linearity for outliers may indicate problems for the measure of maternal height. We estimated SPS excluding outliers according to maternal height, i.e., more than 3 or less than -3 SDs from the mean in the analysis sample for each survey (**eFigure 4** in this Supplement).

We estimated maternal height-standardized mean child HAZ (**eFigure 5** in this Supplement). The -2SD height-for-age threshold for stunting is somewhat arbitrary—exposures to infections and undernutrition shift the whole distribution of height-for-age downward (2).

We show estimates after imputing missing information on stunting using multiple imputation chained equations (**eFigure 6** in this Supplement).

We excluded children born to women younger than 25 years old, when full adult height has surely been attained (**eFigure 7** in this Supplement).

We show results for child wasting, another nutrition outcome (**eFigure 8** in this Supplement). Wasting is primarily used as an indicator for acute undernutrition (as opposed to stunting which is used to measures chronic undernutrition) and appears to be less sensitive to maternal height (3). We, therefore, would not expect the differences between maternal height-standardized prevalence of wasting and crude prevalence of wasting, to be as substantial as in the case of stunting.

We show Pearson's correlation coefficients for the relationship of CPS and SPS with country-level child health variables (**eFigure 9** in this Supplement).

References

1. DHS, Wealth Index Construction. *DHS Program* (2019) (February 19, 2019).

2. N. Perumal, D. G. Bassani, D. E. Roth, Use and Misuse of Stunting as a Measure of Child Health. *J. Nutr.* 148, 311–315 (2018).

3. Z. Li, R. Kim, S. Vollmer, S. V. Subramanian, Factors Associated With Child Stunting, Wasting, and Underweight in 35 Low- and Middle-Income Countries. *JAMA Netw. Open* 3, e203386–e203386 (2020).

eFigure 1. Adjusted maternal height-standardized prevalence of stunting

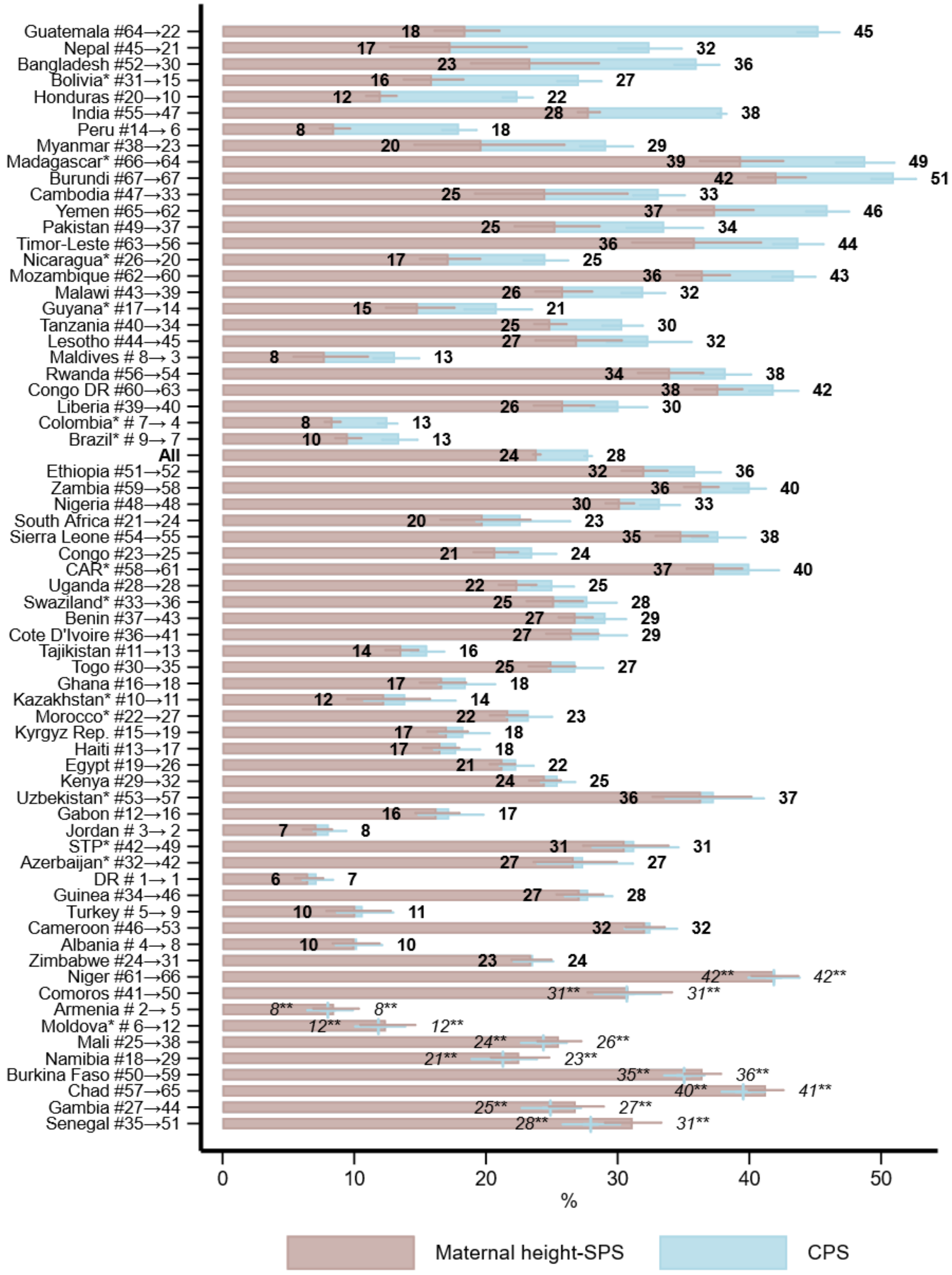

Notes: Countries are ordered (from large to small) according to the difference between crude prevalence of stunting (CPS) and maternal height-standardized prevalence of stunting (SPS). # indicates rank (from low to high) according to CPS and → indicates rank according to SPS. *Indicates estimates from surveys conducted before 2010. **Indicates countries where CPS was higher than SPS. All estimates were weighted using sampling weights which sum up to one for each survey. For SPS, sampling weights were re-scaled to sum up to the probability-density within each stratum, cm of maternal height in the reference population. Standard errors were adjusted for clustering at the PSU-level when estimating CPS, and PSU crossed with maternal height when estimating SPS. 95% confidence intervals are shown. Central African Republic (CAR), Democratic Republic of Congo (Congo DR), Sao Tome and Principe (STP), Dominican Republic (DR), Republic (Rep.). Adjusted maternal height-SPS adjusts for current living standards.

eFigure 2. Prevalence of stunting predicted at the MGRS mean maternal height

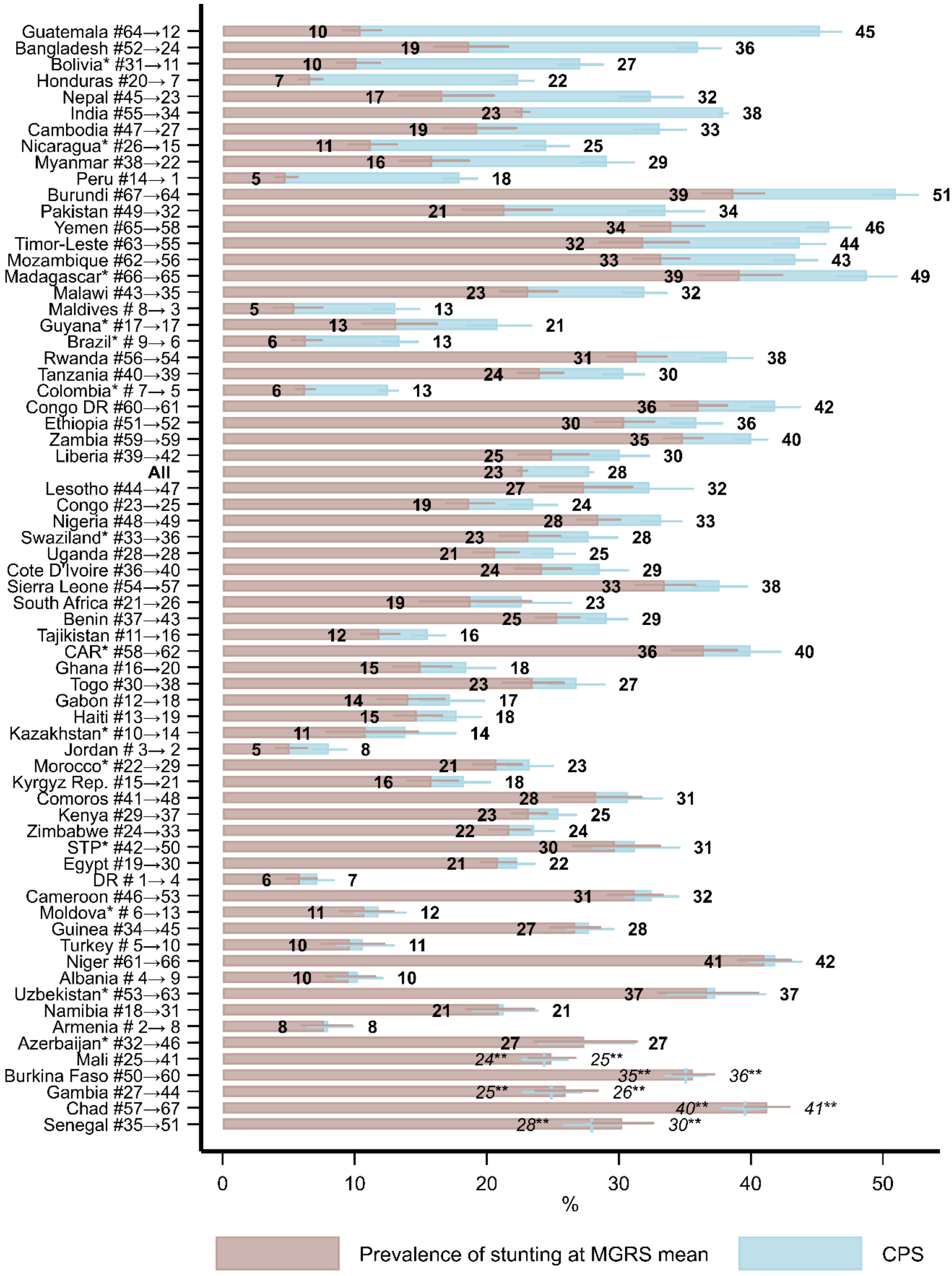

Notes: Countries are ordered (from large to small) according to the difference between crude prevalence of stunting (CPS) and the prevalence of stunting at the MGRS mean. Prevalence of stunting at the MGRS mean was predicted using a logit model of stunting on maternal height. # indicates rank (from low to high) according to CPS and → indicates rank according to predicted stunting. *Indicates estimates from surveys conducted before 2010. **Indicates countries where CPS was higher than predicted stunting. All estimates were weighted using sampling weights which sum up to one for each survey. Standard errors were adjusted for clustering at the PSU-level. 95% confidence intervals are shown. Central African Republic (CAR), Democratic Republic of Congo (Congo DR), Sao Tome and Principe (STP), Dominican Republic (DR), Republic (Rep.).

eFigure 3. Linear fit and LOWESS fit of stunting on maternal height and a histogram of maternal height

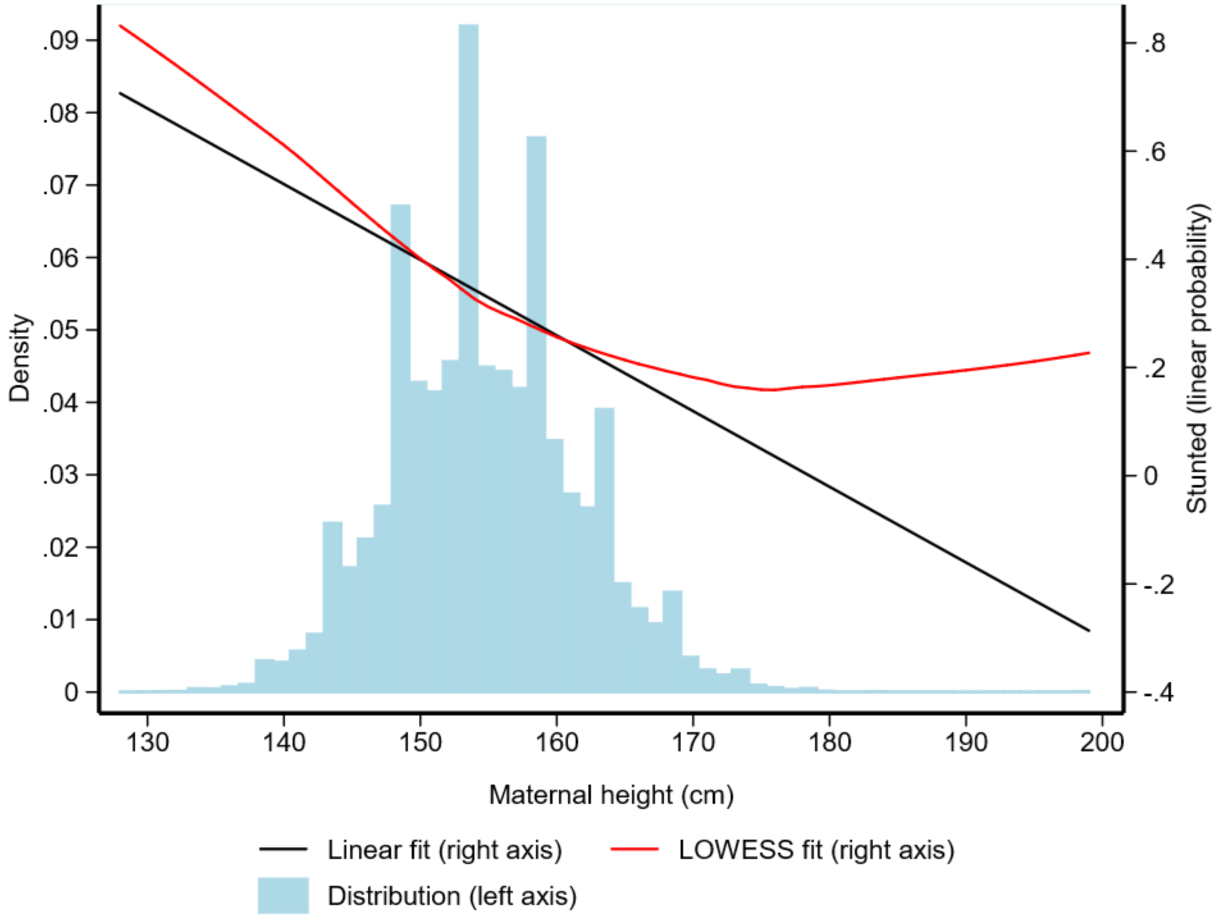

eFigure 4. Maternal height-standardized prevalence of stunting: excluding maternal height outliers

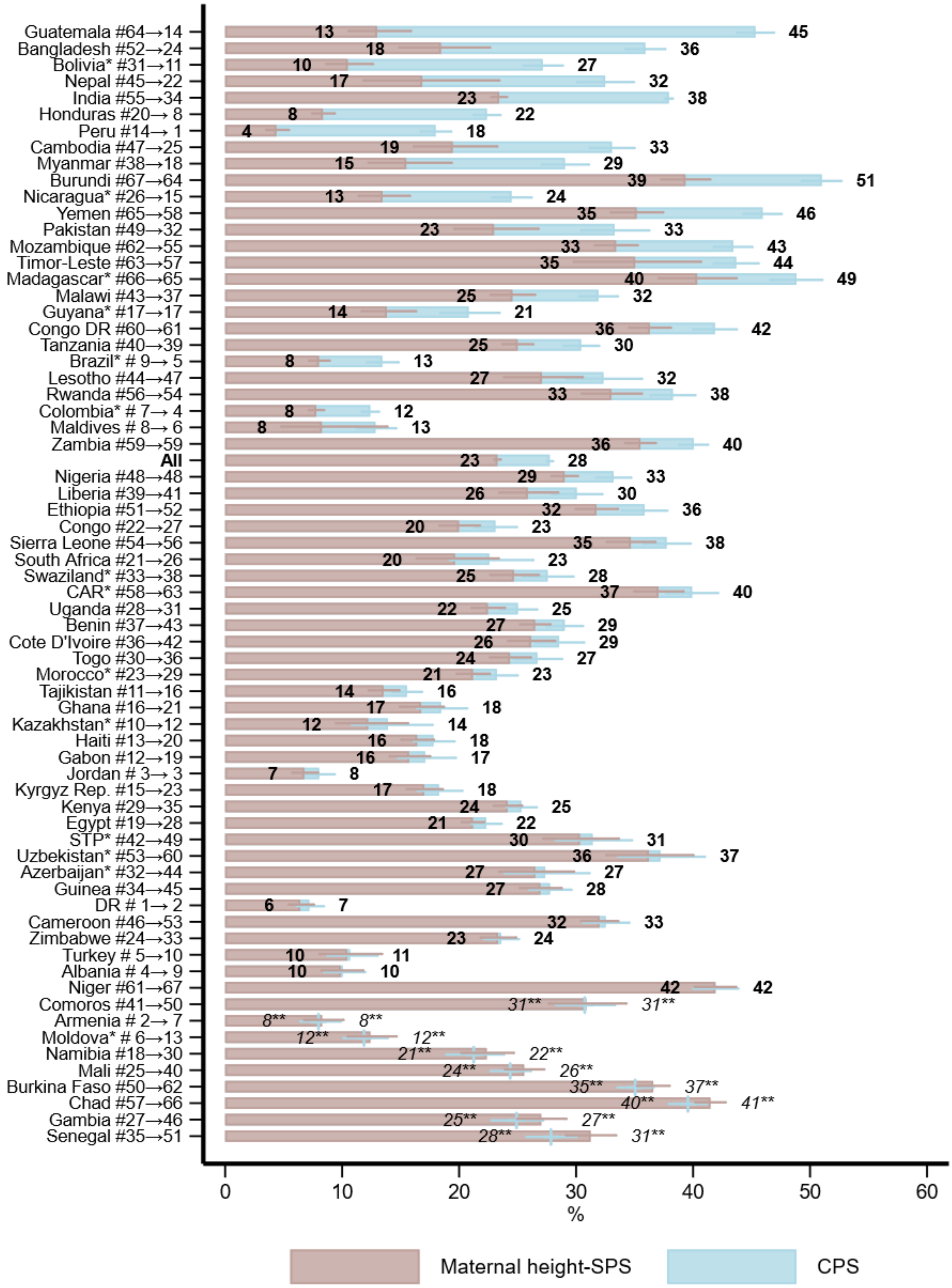

Notes: Countries are ordered (from large to small) according to the difference between crude prevalence of stunting (CPS) and maternal height-standardized prevalence of stunting (SPS). # indicates rank (from low to high) according to CPS and → indicates rank according to SPS. *Indicates estimates from surveys conducted before 2010. ** Indicates countries where CPS was higher than SPS. All estimates were weighted using sampling weights which sum up to one for each survey. For SPS, sampling weights were re-scaled to sum up to the probability-density within each stratum, cm of maternal height in the reference population. Standard errors were adjusted for clustering at the PSU-level when estimating CPS, and PSU crossed with maternal height when estimating SPS. 95% confidence intervals are shown. Central African Republic (CAR), Democratic Republic of Congo (Congo DR), Sao Tome and Principe (STP), Dominican Republic (DR), Republic (Rep.). Outliers with maternal height above 3 and below -3 standard deviations from the mean in each country are excluded.

eFigure 5. Maternal height-standardized mean child height-for-age z-score

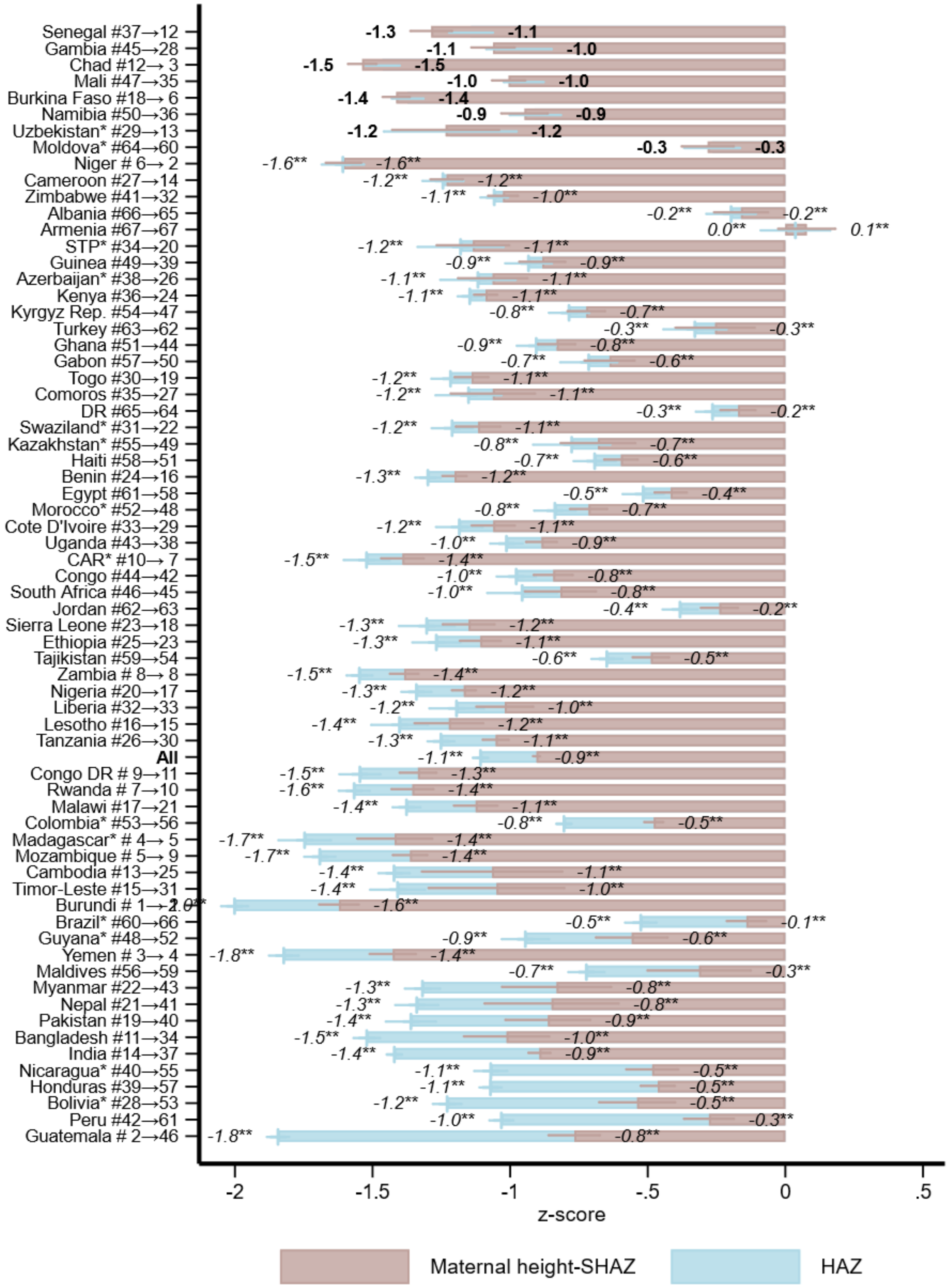

Notes: Countries are ordered (from large to small) according to the difference between mean Height-for-age z-score (HAZ) and maternal height-standardized height-for-age z-score (SHAZ). # indicates rank (from low to high) according to HAZ and → indicates rank according to SHAZ. *Indicates estimates from surveys conducted before 2010. **HAZ higher than SHAZ. All estimates were weighted using sampling weights which sum up to one for each survey. For SHAZ, sampling weights were re-scaled to sum up to the probability-density within each stratum, cm of maternal height in the reference population. Standard errors were adjusted for clustering at the PSU-level when estimating HAZ, and PSU crossed with maternal height when estimating SHAZ. 95% confidence intervals are shown. Central African Republic (CAR), Democratic Republic of Congo (Congo DR), Sao Tome and Principe (STP), Dominican Republic (DR), Republic (Rep.).

eFigure 6 Maternal height-standardized prevalence of stunting: imputing missing stunting information

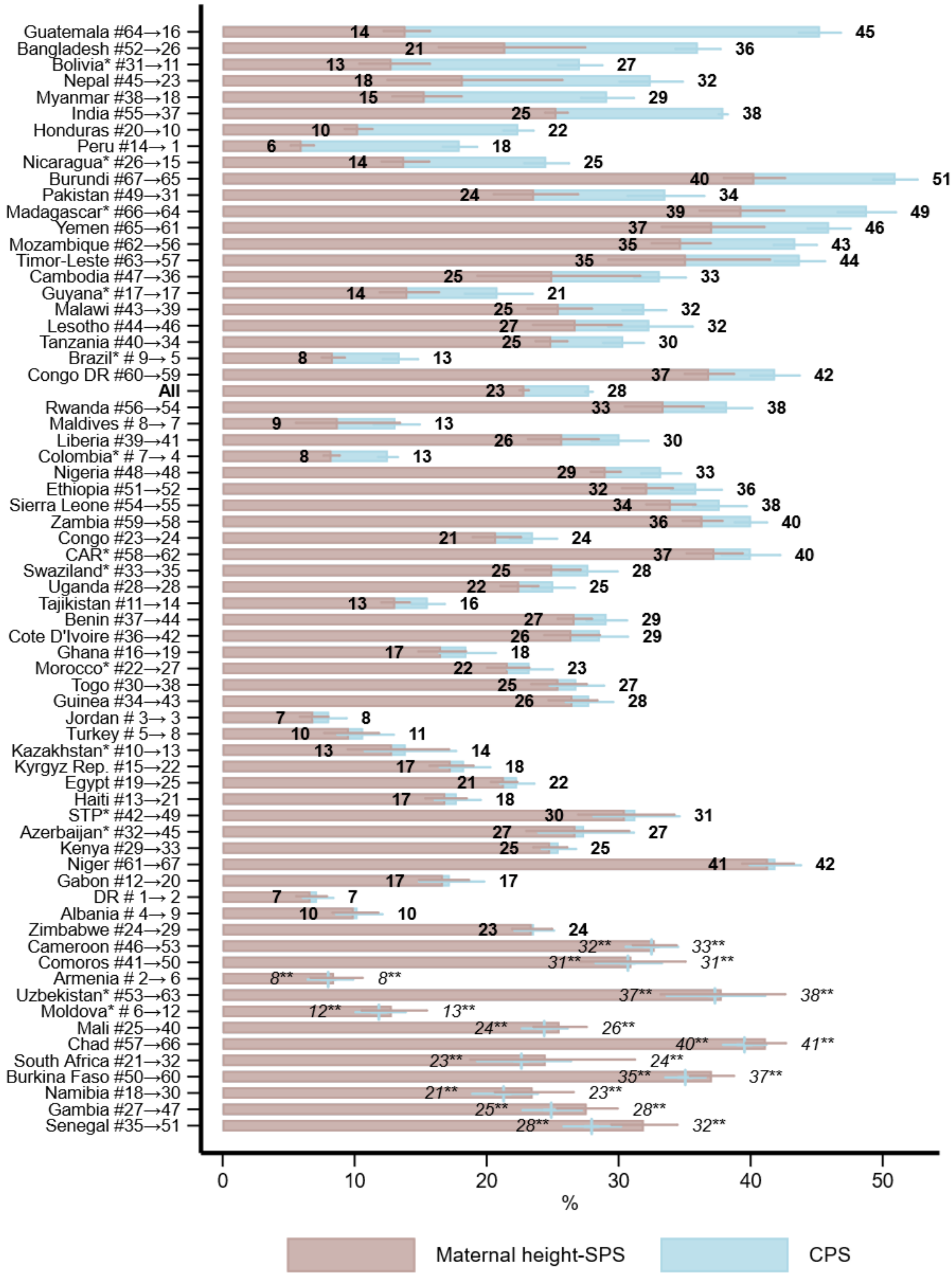

Notes: Countries are ordered (from large to small) according to the difference between crude prevalence of stunting (CPS) and maternal height-standardized prevalence of stunting (SPS). # indicates rank (from low to high) according to CPS and → indicates rank according to SPS. *Indicates estimates from surveys conducted before 2010. **Indicates countries where CPS was higher than SPS. All estimates were weighted using sampling weights which sum up to one for each survey. For SPS, sampling weights were re-scaled to sum up to the probability-density within each stratum, cm of maternal height in the reference population. Standard errors were adjusted for clustering at the PSU-level when estimating CPS, and PSU crossed with maternal height when estimating SPS. 95% confidence intervals are shown. Central African Republic (CAR), Democratic Republic of Congo (Congo DR), Sao Tome and Principe (STP), Dominican Republic (DR), Republic (Rep.). Estimates were obtained from 10 imputations with 10 burn-in iterations. Augmented logit regressions were used for discrete variables and augmented ordinary least squares regressions for continuous variables. Imputations were done using stunting and maternal height, as well as auxiliary variables. Auxiliary variables were: PSU-level mean for child height-for-age z-score (only PSUs with at least two children); and survey-level means for maternal height, household wealth, and child height-for-age z-score.

eFigure 7. Maternal height-standardized prevalence of stunting: excluding children born to mothers younger than 25 years

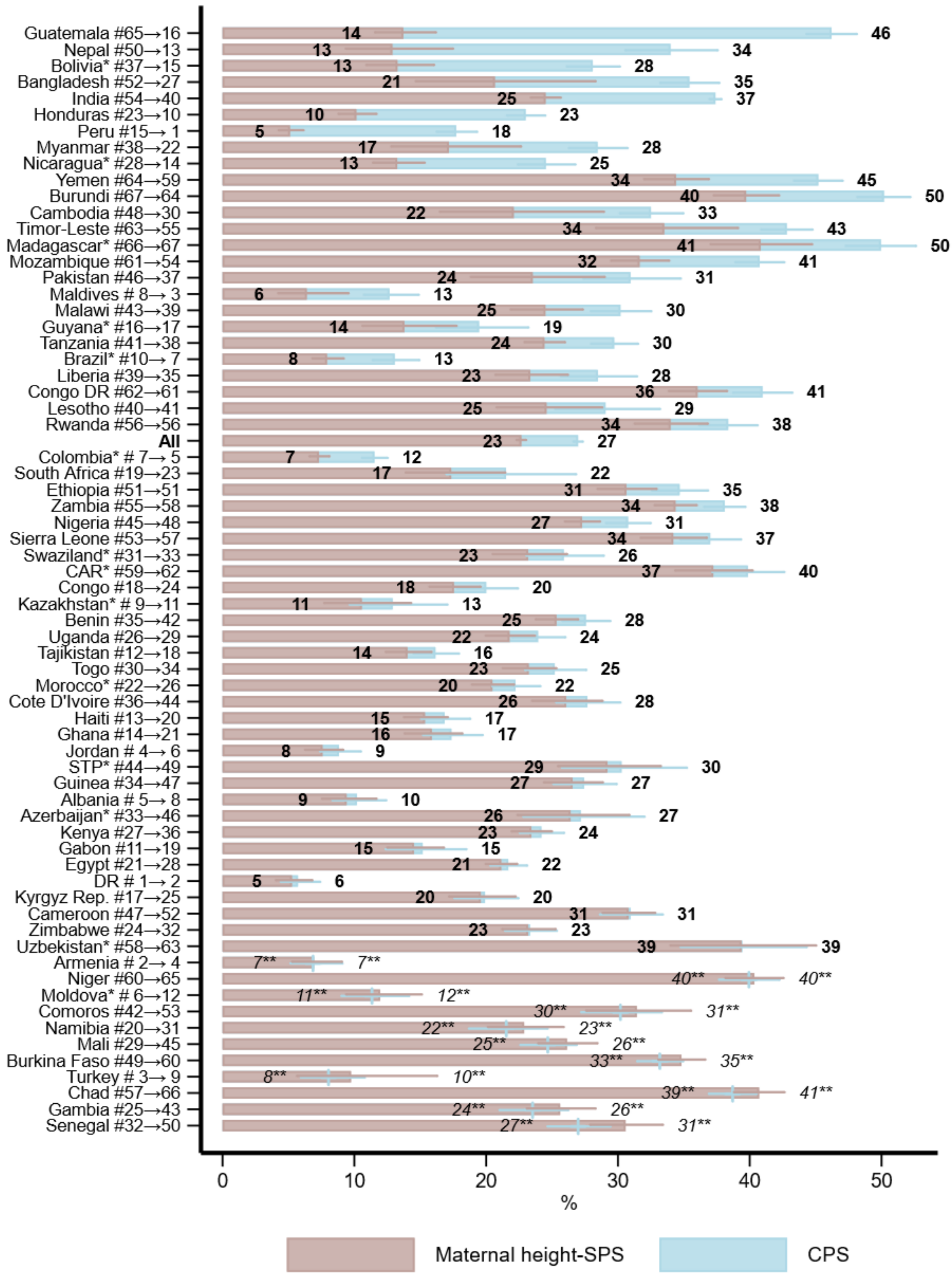

Notes: Countries are ordered (from large to small) according to the difference between crude prevalence of stunting (CPS) and maternal height-standardized prevalence of stunting (SPS). # indicates rank (from low to high) according to CPS and → indicates rank according to SPS. *Indicates estimates from surveys conducted before 2010. **Indicates countries where CPS was higher than SPS. All estimates were weighted using sampling weights which sum up to one for each survey. For SPS, sampling weights were re-scaled to sum up to the probability-density within each stratum, cm of maternal height in the reference population. Standard errors were adjusted for clustering at the PSU-level when estimating CPS, and PSU crossed with maternal height when estimating SPS. 95% confidence intervals are shown. Central African Republic (CAR), Democratic Republic of Congo (Congo DR), Sao Tome and Principe (STP), Dominican Republic (DR), Republic (Rep.).

eFigure 8. Crude and maternal height-standardized prevalence of wasting

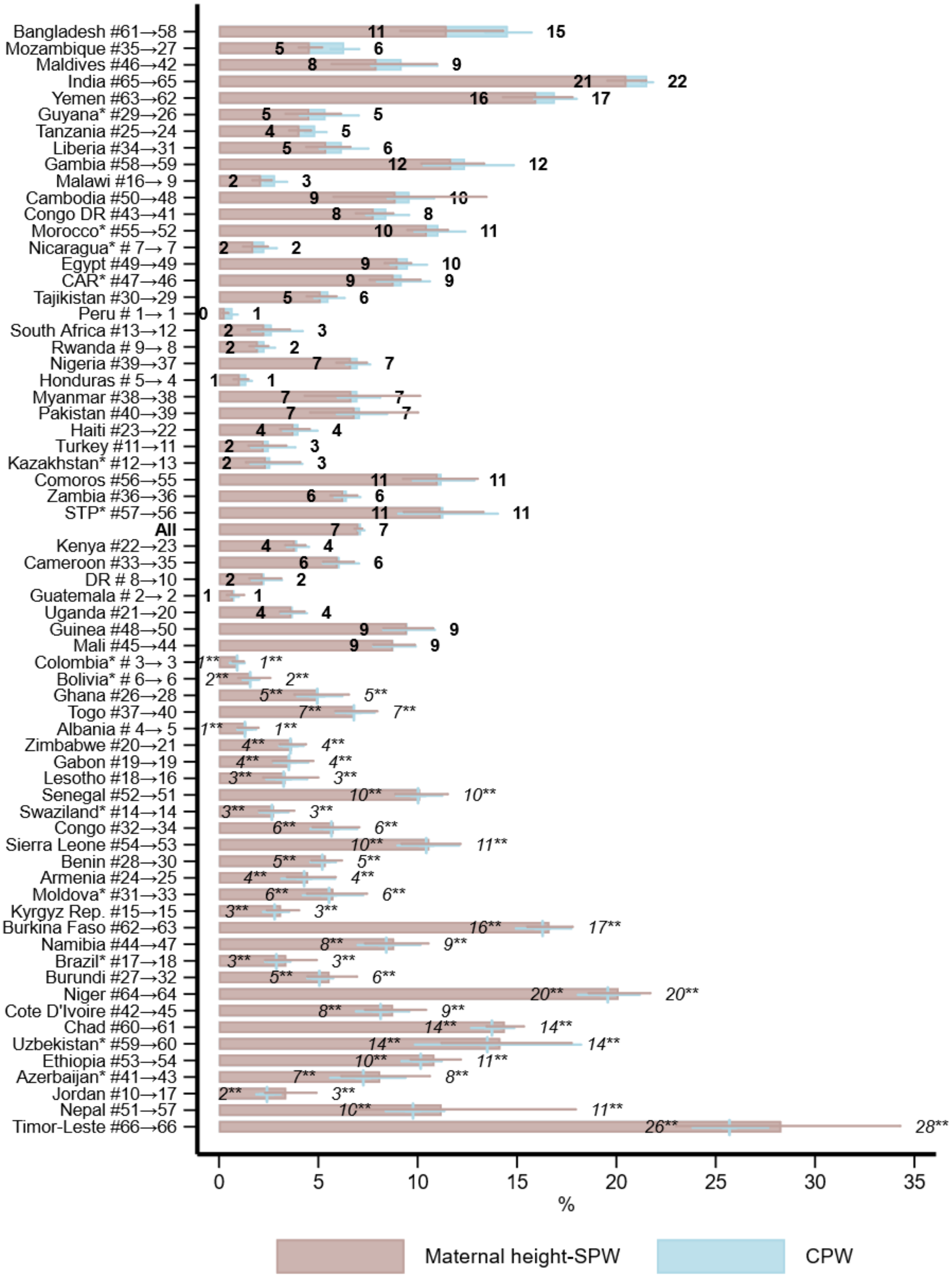

Notes: Countries are ordered (from large to small) according to the difference between crude prevalence of wasting (CPW) and maternal height-standardized prevalence of wasting (SPW). # indicates rank (from low to high) according to CPW and → indicates rank according to SPW. *Indicates estimates from surveys conducted before 2010. **Indicate countries where CPW was higher than SPW. All estimates were weighted using sampling weights which sum up to one for each survey. For SPW, sampling weights were re-scaled to sum up to the probability-density within each stratum, cm of maternal height in the reference population. Standard errors were adjusted for clustering at the PSU-level when estimating CPW, and PSU crossed with maternal height when estimating SPW. 95% confidence intervals are shown. Central African Republic (CAR), Democratic Republic of Congo (Congo DR), Sao Tome and Principe (STP), Dominican Republic (DR), Republic (Rep.).

eFigure 9. Scatterplots and Pearson's correlation coefficients for CPS and maternal height-SPS and other aggregate measures of child health

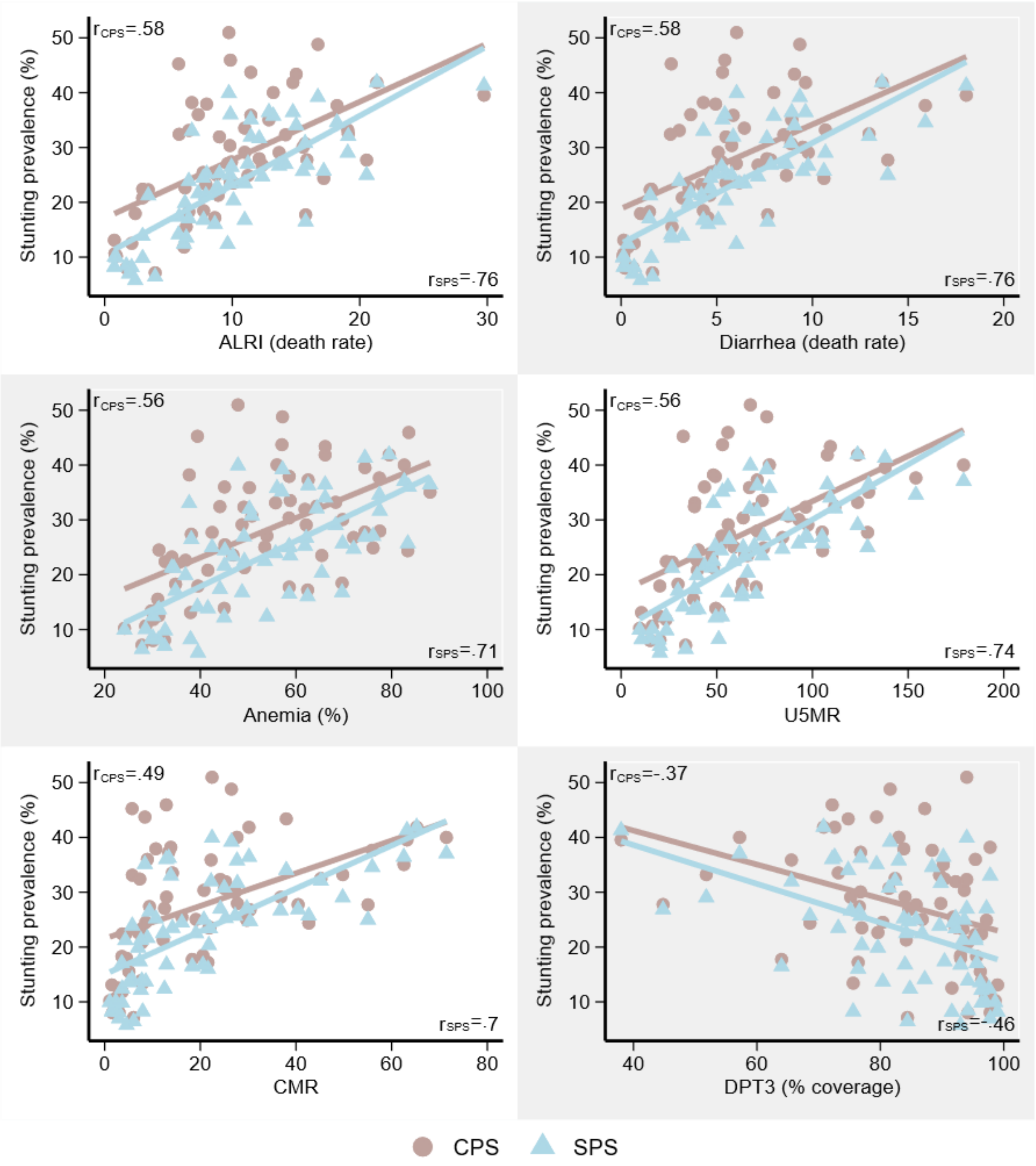

Notes: Pearson's correlation coefficients are shown. Acute lower respiratory infections mortality rate (deaths per 1000 live births) for children under five (ALRI); Diarrhea mortality rate (deaths per 1000 live births) for children under five (Diarrhea); Prevalence of anemia (%) among children under five (Anemia); Under-five mortality rate (deaths per 1000 live births, U5MR); Child mortality rate (deaths of children 1–4 years old per 1000 live births, CMR); Diphtheria-pertussis-tetanus vaccination coverage (%) for children 1 – 2 years old (DPT3).

eAppendix 2. Supplementary analyses

SPS can also be obtained through ordinary least squares (OLS) regression models:

$$y = \alpha + \beta_1 (m - r) + \varepsilon$$

where a binary indicator for stunting (y) is regressed on maternal height (m) centered around the mean height of mothers in the reference population (r), with sampling weights re-scaled to sum up to the probability-density within each stratum of maternal height in the reference population. SPS is given by α which is presented as a percentage ($\alpha \times 100$). Note that since maternal height is centered, α remains the same regardless of whether $\beta_1(m - r)$ is included or not. CPS can be obtained in the same way, by replacing r with the sample mean of maternal height and not re-scaling the sampling weights. β_1 gives the association between maternal height and stunting. We show coefficients for maternal height, both with and without standardization (i.e., re-scaling of sampling weights), in order to confirm the relationship between maternal height and stunting in each country in **eTable 4** in this Supplement.

Standardization according to the height of both parents can also be estimated using OLS as:

$$y = \alpha + \beta_1(m - r) + \beta_2(p - mr) + \varepsilon$$

In addition to maternal height (m) centered around the mean maternal height in the reference population (r), paternal height (p) was centered around the male height of the reference population (mr). As before, α gives the proportion stunted. When standardizing heights of both parents, sampling weights were re-scaled to sum up to the bivariate normal probability-density within each stratum of parental height. The bivariate normal probability-density was estimated using the covariance between the height of mothers and fathers in each target sample, in addition to the means and SDs of parents in the MGRS.

We show sample sizes, missingness, and average height of mothers and fathers for the male sub-sample in **eTable 5**, **eTable 6**, and **eTable 7** in this Supplement. We show parental height-SPS in **eFigure 10** and **eFigure 11** in this Supplement. We also show regression parameters for parental height in **eTable 8** in this supplement. In addition, we show standardization according to paternal height only and paternal height while adjusting for maternal height (as well as SES). We do the same for maternal height using the male sub-sample as well as showing CPS in the male sub-sample.

eTable 4. Results from regression of stunting on maternal height

		Constant		Mother's height		Household wealth	
Country	Estimate	Constant	95% CI	Coefficient	95% CI	Coefficient	95% CI
All	Crude	0.278**	0.275 to 0.281	-0.012**	-0.012, -0.012	NA	NA
All	Standardized	0.233**	0.230 to 0.236	-0.010**	-0.011, -0.010	NA	NA
All	Standardized (adjusted)	0.239**	0.236 to 0.242	-0.009**	-0.010, -0.009	-0.056**	-0.059, -0.053
Albania	Crude	0.102**	0.085 to 0.120	-0.004**	-0.007, -0.001	NA	NA
Albania	Standardized	0.099**	0.082 to 0.117	-0.003**	-0.007, -0.000	NA	NA
Albania	Standardized (adjusted)	0.100**	0.082 to 0.118	-0.003	-0.006 to 0.001	-0.022**	-0.041, -0.003
Armenia	Crude	0.080**	0.063 to 0.097	-0.002	-0.005 to 0.001	NA	NA
Armenia	Standardized	0.085**	0.067 to 0.102	-0.002	-0.005 to 0.002	NA	NA
Armenia	Standardized (adjusted)	0.085**	0.067 to 0.102	-0.002	-0.005 to 0.002	-0.022**	-0.041, -0.004
Azerbaijan	Crude	0.274**	0.238 to 0.310	-0.000	-0.005 to 0.004	NA	NA
Azerbaijan	Standardized	0.265**	0.233 to 0.296	-0.001	-0.007 to 0.005	NA	NA
Azerbaijan	Standardized (adjusted)	0.267**	0.235 to 0.298	-0.001	-0.007 to 0.005	-0.057**	-0.091, -0.024
Bangladesh	Crude	0.360**	0.343 to 0.377	-0.018**	-0.022, -0.015	NA	NA
Bangladesh	Standardized	0.216**	0.168 to 0.265	-0.010**	-0.017, -0.002	NA	NA
Bangladesh	Standardized (adjusted)	0.234**	0.185 to 0.282	-0.008**	-0.015, -0.001	-0.102**	-0.137, -0.066
Benin	Crude	0.291**	0.275 to 0.306	-0.014**	-0.016, -0.012	NA	NA
Benin	Standardized	0.267**	0.254 to 0.280	-0.013**	-0.015, -0.010	NA	NA
Benin	Standardized (adjusted)	0.268**	0.255 to 0.281	-0.012**	-0.014, -0.010	-0.076**	-0.088, -0.064
Bolivia	Crude	0.271**	0.254 to 0.287	-0.020**	-0.023, -0.018	NA	NA
Bolivia	Standardized	0.124**	0.103 to 0.145	-0.012**	-0.015, -0.009	NA	NA

		Constant		Mother's height		Household wealth	
Country	Estimate	Constant	95% CI	Coefficient	95% CI	Coefficient	95% CI
Bolivia	Standardized (adjusted)	0.159**	0.136 to 0.182	-0.008**	-0.012, -0.005	-0.064**	-0.082, -0.046
Brazil	Crude	0.134**	0.121 to 0.147	-0.013**	-0.015, -0.011	NA	NA
Brazil	Standardized	0.082**	0.073 to 0.091	-0.010**	-0.011, -0.009	NA	NA
Brazil	Standardized (adjusted)	0.095**	0.085 to 0.105	-0.007**	-0.008, -0.006	-0.061**	-0.072, -0.050
Burkina Faso	Crude	0.350**	0.335 to 0.366	-0.012**	-0.015, -0.010	NA	NA
Burkina Faso	Standardized	0.364**	0.350 to 0.379	-0.013**	-0.016, -0.011	NA	NA
Burkina Faso	Standardized (adjusted)	0.364**	0.351 to 0.378	-0.012**	-0.015, -0.010	-0.086**	-0.101, -0.071
Burundi	Crude	0.510**	0.493 to 0.527	-0.021**	-0.023, -0.019	NA	NA
Burundi	Standardized	0.399**	0.378 to 0.421	-0.019**	-0.023, -0.016	NA	NA
Burundi	Standardized (adjusted)	0.421**	0.399 to 0.443	-0.015**	-0.019, -0.011	-0.086**	-0.112, -0.060
Cambodia	Crude	0.331**	0.311 to 0.351	-0.018**	-0.022, -0.015	NA	NA
Cambodia	Standardized	0.239**	0.184 to 0.294	-0.007	-0.018 to 0.003	NA	NA
Cambodia	Standardized (adjusted)	0.245**	0.187 to 0.303	-0.007	-0.017 to 0.004	-0.041*	-0.087 to 0.005
Cameroon	Crude	0.325**	0.305 to 0.345	-0.013**	-0.016, -0.010	NA	NA
Cameroon	Standardized	0.321**	0.305 to 0.337	-0.013**	-0.015, -0.011	NA	NA
Cameroon	Standardized (adjusted)	0.321**	0.306 to 0.336	-0.012**	-0.014, -0.009	-0.127**	-0.141, -0.113
Central African Republic	Crude	0.402**	0.379 to 0.424	-0.013**	-0.016, -0.010	NA	NA
Central African Republic	Standardized	0.375**	0.354 to 0.397	-0.012**	-0.015, -0.009	NA	NA
Central African Republic	Standardized (adjusted)	0.378**	0.356 to 0.399	-0.011**	-0.014, -0.008	-0.036**	-0.052, -0.020
Chad	Crude	0.395**	0.379 to 0.412	-0.011**	-0.013, -0.008	NA	NA
Chad	Standardized	0.413**	0.400 to 0.426	-0.010**	-0.012, -0.008	NA	NA

		Constant		Mother's height		Household wealth	
Country	Estimate	Constant	95% CI	Coefficient	95% CI	Coefficient	95% CI
Chad	Standardized (adjusted)	0.413**	0.399 to 0.426	-0.010**	-0.012, -0.008	-0.033**	-0.045, -0.020
Colombia	Crude	0.125**	0.118 to 0.133	-0.012**	-0.013, -0.011	NA	NA
Colombia	Standardized	0.082**	0.076 to 0.088	-0.008**	-0.009, -0.007	NA	NA
Colombia	Standardized (adjusted)	0.083**	0.077 to 0.089	-0.008**	-0.009, -0.007	-0.028**	-0.033, -0.022
Comoros	Crude	0.307**	0.282 to 0.332	-0.006**	-0.011, -0.001	NA	NA
Comoros	Standardized	0.308**	0.275 to 0.341	0.001	-0.005 to 0.006	NA	NA
Comoros	Standardized (adjusted)	0.308**	0.276 to 0.340	0.001	-0.005 to 0.006	-0.062**	-0.098, -0.026
Congo	Crude	0.235**	0.217 to 0.253	-0.016**	-0.019, -0.012	NA	NA
Congo	Standardized	0.203**	0.186 to 0.221	-0.015**	-0.017, -0.012	NA	NA
Congo	Standardized (adjusted)	0.207**	0.190 to 0.224	-0.013**	-0.016, -0.010	-0.063**	-0.077, -0.050
Congo Democratic Republic	Crude	0.418**	0.400 to 0.437	-0.013**	-0.016, -0.011	NA	NA
Congo Democratic Republic	Standardized	0.364**	0.347 to 0.382	-0.012**	-0.015, -0.009	NA	NA
Congo Democratic Republic	Standardized (adjusted)	0.376**	0.358 to 0.395	-0.010**	-0.012, -0.007	-0.091**	-0.105, -0.076
Cote D'Ivoire	Crude	0.286**	0.265 to 0.307	-0.015**	-0.019, -0.012	NA	NA
Cote D'Ivoire	Standardized	0.262**	0.242 to 0.282	-0.013**	-0.017, -0.010	NA	NA
Cote D'Ivoire	Standardized (adjusted)	0.265**	0.245 to 0.285	-0.012**	-0.015, -0.008	-0.065**	-0.087, -0.043
Dominican Republic	Crude	0.071**	0.060 to 0.083	-0.004**	-0.006, -0.003	NA	NA
Dominican Republic	Standardized	0.064**	0.054 to 0.075	-0.004**	-0.006, -0.003	NA	NA
Dominican Republic	Standardized (adjusted)	0.065**	0.054 to 0.076	-0.004**	-0.006, -0.003	-0.029**	-0.040, -0.017
Egypt	Crude	0.223**	0.210 to 0.236	-0.007**	-0.009, -0.006	NA	NA
Egypt	Standardized	0.212**	0.203 to 0.222	-0.007**	-0.009, -0.005	NA	NA

		Constant		Mother's height		Household wealth	
Country	Estimate	Constant	95% CI	Coefficient	95% CI	Coefficient	95% CI
Egypt	Standardized (adjusted)	0.212**	0.203 to 0.222	-0.007**	-0.009, -0.005	0.001	-0.009 to 0.011
Ethiopia	Crude	0.359**	0.339 to 0.378	-0.014**	-0.016, -0.011	NA	NA
Ethiopia	Standardized	0.320**	0.302 to 0.338	-0.010**	-0.013, -0.007	NA	NA
Ethiopia	Standardized (adjusted)	0.320**	0.303 to 0.338	-0.009**	-0.012, -0.007	-0.085**	-0.098, -0.071
Gabon	Crude	0.172**	0.147 to 0.197	-0.013**	-0.015, -0.010	NA	NA
Gabon	Standardized	0.160**	0.143 to 0.178	-0.013**	-0.015, -0.010	NA	NA
Gabon	Standardized (adjusted)	0.162**	0.146 to 0.179	-0.009**	-0.012, -0.007	-0.075**	-0.091, -0.059
Gambia	Crude	0.249**	0.226 to 0.272	-0.012**	-0.016, -0.008	NA	NA
Gambia	Standardized	0.271**	0.250 to 0.292	-0.014**	-0.017, -0.010	NA	NA
Gambia	Standardized (adjusted)	0.268**	0.247 to 0.289	-0.012**	-0.016, -0.009	-0.055**	-0.079, -0.031
Ghana	Crude	0.185**	0.163 to 0.206	-0.013**	-0.016, -0.009	NA	NA
Ghana	Standardized	0.168**	0.149 to 0.186	-0.010**	-0.014, -0.007	NA	NA
Ghana	Standardized (adjusted)	0.167**	0.149 to 0.184	-0.010**	-0.014, -0.007	-0.064**	-0.080, -0.048
Guatemala	Crude	0.452**	0.436 to 0.468	-0.032**	-0.034, -0.030	NA	NA
Guatemala	Standardized	0.141**	0.125 to 0.158	-0.019**	-0.020, -0.017	NA	NA
Guatemala	Standardized (adjusted)	0.184**	0.160 to 0.209	-0.015**	-0.018, -0.013	-0.058**	-0.079, -0.038
Guinea	Crude	0.278**	0.260 to 0.296	-0.006**	-0.009, -0.003	NA	NA
Guinea	Standardized	0.269**	0.251 to 0.286	-0.005**	-0.008, -0.001	NA	NA
Guinea	Standardized (adjusted)	0.271**	0.253 to 0.289	-0.003*	-0.006 to 0.000	-0.053**	-0.072, -0.034
Guyana	Crude	0.208**	0.182 to 0.234	-0.013**	-0.018, -0.009	NA	NA
Guyana	Standardized	0.138**	0.115 to 0.162	-0.011**	-0.014, -0.008	NA	NA

		Constant		Mother's height		Household wealth	
Country	Estimate	Constant	95% CI	Coefficient	95% CI	Coefficient	95% CI
Guyana	Standardized (adjusted)	0.148**	0.122 to 0.174	-0.010**	-0.013, -0.006	-0.034**	-0.061, -0.006
Haiti	Crude	0.177**	0.160 to 0.195	-0.012**	-0.014, -0.010	NA	NA
Haiti	Standardized	0.165**	0.151 to 0.179	-0.012**	-0.014, -0.010	NA	NA
Haiti	Standardized (adjusted)	0.165**	0.152 to 0.179	-0.011**	-0.014, -0.009	-0.065**	-0.078, -0.052
Honduras	Crude	0.224**	0.212 to 0.235	-0.022**	-0.024, -0.021	NA	NA
Honduras	Standardized	0.098**	0.089 to 0.108	-0.012**	-0.013, -0.011	NA	NA
Honduras	Standardized (adjusted)	0.120**	0.108 to 0.132	-0.009**	-0.011, -0.008	-0.062**	-0.074, -0.051
India	Crude	0.379**	0.376 to 0.383	-0.017**	-0.017, -0.016	NA	NA
India	Standardized	0.252**	0.244 to 0.260	-0.010**	-0.011, -0.008	NA	NA
India	Standardized (adjusted)	0.278**	0.270 to 0.287	-0.008**	-0.009, -0.006	-0.076**	-0.084, -0.067
Jordan	Crude	0.081**	0.068 to 0.093	-0.009**	-0.011, -0.006	NA	NA
Jordan	Standardized	0.070**	0.059 to 0.081	-0.007**	-0.009, -0.005	NA	NA
Jordan	Standardized (adjusted)	0.071**	0.060 to 0.082	-0.007**	-0.008, -0.005	-0.024**	-0.039, -0.008
Kazakhstan	Crude	0.139**	0.104 to 0.173	-0.011**	-0.016, -0.006	NA	NA
Kazakhstan	Standardized	0.124**	0.092 to 0.155	-0.012**	-0.017, -0.006	NA	NA
Kazakhstan	Standardized (adjusted)	0.124**	0.092 to 0.156	-0.011**	-0.017, -0.006	-0.007	-0.035 to 0.022
Kenya	Crude	0.255**	0.241 to 0.268	-0.012**	-0.014, -0.010	NA	NA
Kenya	Standardized	0.244**	0.232 to 0.257	-0.012**	-0.014, -0.010	NA	NA
Kenya	Standardized (adjusted)	0.245**	0.232 to 0.257	-0.011**	-0.013, -0.009	-0.060**	-0.071, -0.048
Kyrgyz Republic	Crude	0.183**	0.164 to 0.202	-0.011**	-0.014, -0.008	NA	NA
Kyrgyz Republic	Standardized	0.171**	0.155 to 0.186	-0.009**	-0.012, -0.007	NA	NA

		Constant		Mother's height		Household wealth	
Country	Estimate	Constant	95% CI	Coefficient	95% CI	Coefficient	95% CI
Kyrgyz Republic	Standardized (adjusted)	0.170**	0.155 to 0.186	-0.010**	-0.012, -0.007	0.008	-0.011 to 0.028
Lesotho	Crude	0.323**	0.291 to 0.355	-0.012**	-0.017, -0.007	NA	NA
Lesotho	Standardized	0.270**	0.237 to 0.302	-0.014**	-0.019, -0.009	NA	NA
Lesotho	Standardized (adjusted)	0.269**	0.236 to 0.302	-0.014**	-0.018, -0.009	-0.076**	-0.104, -0.047
Liberia	Crude	0.301**	0.279 to 0.322	-0.012**	-0.015, -0.009	NA	NA
Liberia	Standardized	0.257**	0.233 to 0.280	-0.011**	-0.015, -0.008	NA	NA
Liberia	Standardized (adjusted)	0.259**	0.236 to 0.282	-0.011**	-0.014, -0.008	-0.048**	-0.070, -0.027
Madagascar	Crude	0.488**	0.466 to 0.510	-0.012**	-0.015, -0.009	NA	NA
Madagascar	Standardized	0.392**	0.361 to 0.423	-0.013**	-0.018, -0.008	NA	NA
Madagascar	Standardized (adjusted)	0.394**	0.362 to 0.425	-0.013**	-0.018, -0.008	-0.023	-0.052 to 0.006
Malawi	Crude	0.319**	0.303 to 0.336	-0.017**	-0.020, -0.015	NA	NA
Malawi	Standardized	0.253**	0.232 to 0.274	-0.013**	-0.017, -0.009	NA	NA
Malawi	Standardized (adjusted)	0.259**	0.237 to 0.280	-0.012**	-0.016, -0.008	-0.053**	-0.073, -0.033
Maldives	Crude	0.131**	0.114 to 0.148	-0.011**	-0.014, -0.007	NA	NA
Maldives	Standardized	0.082**	0.050 to 0.114	-0.003	-0.008 to 0.002	NA	NA
Maldives	Standardized (adjusted)	0.078**	0.050 to 0.105	-0.004*	-0.008 to 0.001	-0.041**	-0.077, -0.004
Mali	Crude	0.244**	0.226 to 0.261	-0.009**	-0.011, -0.006	NA	NA
Mali	Standardized	0.257**	0.240 to 0.274	-0.007**	-0.010, -0.004	NA	NA
Mali	Standardized (adjusted)	0.255**	0.239 to 0.272	-0.005**	-0.009, -0.002	-0.068**	-0.084, -0.052
Moldova	Crude	0.118**	0.099 to 0.137	-0.010**	-0.014, -0.007	NA	NA
Moldova	Standardized	0.124**	0.103 to 0.145	-0.010**	-0.014, -0.006	NA	NA

		Constant		Mother's height		Household wealth	
Country	Estimate	Constant	95% CI	Coefficient	95% CI	Coefficient	95% CI
Moldova	Standardized (adjusted)	0.124**	0.103 to 0.145	-0.009**	-0.014, -0.005	-0.024**	-0.041, -0.007
Morocco	Crude	0.232**	0.215 to 0.250	-0.009**	-0.011, -0.006	NA	NA
Morocco	Standardized	0.213**	0.198 to 0.227	-0.009**	-0.011, -0.006	NA	NA
Morocco	Standardized (adjusted)	0.217**	0.203 to 0.231	-0.007**	-0.010, -0.005	-0.067**	-0.080, -0.053
Mozambique	Crude	0.434**	0.417 to 0.450	-0.016**	-0.018, -0.014	NA	NA
Mozambique	Standardized	0.340**	0.322 to 0.359	-0.014**	-0.017, -0.010	NA	NA
Mozambique	Standardized (adjusted)	0.365**	0.344 to 0.385	-0.010**	-0.013, -0.006	-0.076**	-0.093, -0.059
Myanmar	Crude	0.291**	0.271 to 0.311	-0.017**	-0.020, -0.014	NA	NA
Myanmar	Standardized	0.168**	0.122 to 0.214	-0.012**	-0.022, -0.002	NA	NA
Myanmar	Standardized (adjusted)	0.196**	0.140 to 0.253	-0.008	-0.019 to 0.002	-0.068**	-0.114, -0.023
Namibia	Crude	0.213**	0.188 to 0.238	-0.011**	-0.015, -0.008	NA	NA
Namibia	Standardized	0.225**	0.203 to 0.248	-0.012**	-0.016, -0.009	NA	NA
Namibia	Standardized (adjusted)	0.225**	0.203 to 0.247	-0.012**	-0.016, -0.008	-0.072**	-0.095, -0.050
Nepal	Crude	0.324**	0.300 to 0.348	-0.018**	-0.022, -0.014	NA	NA
Nepal	Standardized	0.174**	0.124 to 0.224	-0.014**	-0.021, -0.007	NA	NA
Nepal	Standardized (adjusted)	0.173**	0.121 to 0.225	-0.015**	-0.022, -0.007	-0.052**	-0.088, -0.017
Nicaragua	Crude	0.245**	0.228 to 0.262	-0.018**	-0.021, -0.015	NA	NA
Nicaragua	Standardized	0.137**	0.120 to 0.154	-0.013**	-0.015, -0.011	NA	NA
Nicaragua	Standardized (adjusted)	0.172**	0.149 to 0.194	-0.007**	-0.010, -0.004	-0.089**	-0.112, -0.065
Niger	Crude	0.419**	0.399 to 0.438	-0.013**	-0.016, -0.010	NA	NA
Niger	Standardized	0.419**	0.401 to 0.437	-0.013**	-0.016, -0.010	NA	NA

		Constant		Mother's height		Household wealth	
Country	Estimate	Constant	95% CI	Coefficient	95% CI	Coefficient	95% CI
Niger	Standardized (adjusted)	0.419**	0.402 to 0.437	-0.012**	-0.015, -0.009	-0.050**	-0.064, -0.035
Nigeria	Crude	0.332**	0.317 to 0.347	-0.016**	-0.018, -0.014	NA	NA
Nigeria	Standardized	0.290**	0.279 to 0.302	-0.016**	-0.018, -0.014	NA	NA
Nigeria	Standardized (adjusted)	0.302**	0.291 to 0.312	-0.012**	-0.013, -0.010	-0.122**	-0.133, -0.111
Pakistan	Crude	0.335**	0.306 to 0.364	-0.019**	-0.022, -0.015	NA	NA
Pakistan	Standardized	0.235**	0.205 to 0.265	-0.014**	-0.019, -0.010	NA	NA
Pakistan	Standardized (adjusted)	0.253**	0.220 to 0.285	-0.011**	-0.016, -0.007	-0.086**	-0.116, -0.056
Peru	Crude	0.179**	0.166 to 0.193	-0.018**	-0.020, -0.016	NA	NA
Peru	Standardized	0.058**	0.050 to 0.066	-0.010**	-0.012, -0.009	NA	NA
Peru	Standardized (adjusted)	0.085**	0.073 to 0.097	-0.007**	-0.009, -0.006	-0.050**	-0.061, -0.038
Rwanda	Crude	0.382**	0.363 to 0.401	-0.016**	-0.018, -0.013	NA	NA
Rwanda	Standardized	0.330**	0.306 to 0.355	-0.013**	-0.018, -0.008	NA	NA
Rwanda	Standardized (adjusted)	0.340**	0.315 to 0.365	-0.010**	-0.016, -0.005	-0.080**	-0.100, -0.060
Sao Tome & Principe	Crude	0.313**	0.280 to 0.345	-0.009**	-0.013, -0.004	NA	NA
Sao Tome & Principe	Standardized	0.303**	0.270 to 0.335	-0.011**	-0.016, -0.006	NA	NA
Sao Tome & Principe	Standardized (adjusted)	0.305**	0.273 to 0.338	-0.010**	-0.015, -0.005	-0.046**	-0.075, -0.017
Senegal	Crude	0.280**	0.258 to 0.301	-0.010**	-0.013, -0.008	NA	NA
Senegal	Standardized	0.316**	0.295 to 0.338	-0.013**	-0.016, -0.009	NA	NA
Senegal	Standardized (adjusted)	0.311**	0.290 to 0.333	-0.011**	-0.015, -0.007	-0.082**	-0.104, -0.059
Sierra Leone	Crude	0.377**	0.356 to 0.397	-0.011**	-0.014, -0.008	NA	NA
Sierra Leone	Standardized	0.346**	0.325 to 0.366	-0.009**	-0.013, -0.006	NA	NA

		Constant		Mother's height		Household wealth	
Country	Estimate	Constant	95% CI	Coefficient	95% CI	Coefficient	95% CI
Sierra Leone	Standardized (adjusted)	0.348**	0.328 to 0.368	-0.009**	-0.012, -0.006	-0.032**	-0.061, -0.004
South Africa	Crude	0.226**	0.191 to 0.262	-0.012**	-0.018, -0.006	NA	NA
South Africa	Standardized	0.199**	0.164 to 0.234	-0.007**	-0.013, -0.000	NA	NA
South Africa	Standardized (adjusted)	0.197**	0.163 to 0.232	-0.007**	-0.013, -0.001	-0.065**	-0.100, -0.030
Swaziland	Crude	0.277**	0.255 to 0.299	-0.016**	-0.020, -0.012	NA	NA
Swaziland	Standardized	0.250**	0.228 to 0.271	-0.015**	-0.019, -0.012	NA	NA
Swaziland	Standardized (adjusted)	0.252**	0.230 to 0.273	-0.014**	-0.018, -0.011	-0.052**	-0.074, -0.029
Tajikistan	Crude	0.155**	0.143 to 0.168	-0.010**	-0.012, -0.008	NA	NA
Tajikistan	Standardized	0.136**	0.123 to 0.148	-0.007**	-0.010, -0.005	NA	NA
Tajikistan	Standardized (adjusted)	0.136**	0.123 to 0.148	-0.007**	-0.010, -0.005	-0.001	-0.015 to 0.014
Tanzania	Crude	0.304**	0.288 to 0.319	-0.014**	-0.016, -0.012	NA	NA
Tanzania	Standardized	0.250**	0.237 to 0.262	-0.014**	-0.016, -0.012	NA	NA
Tanzania	Standardized (adjusted)	0.249**	0.236 to 0.261	-0.014**	-0.016, -0.012	-0.066**	-0.077, -0.056
Timor-Leste	Crude	0.437**	0.418 to 0.456	-0.013**	-0.016, -0.010	NA	NA
Timor-Leste	Standardized	0.351**	0.302 to 0.400	-0.008**	-0.015, -0.001	NA	NA
Timor-Leste	Standardized (adjusted)	0.359**	0.309 to 0.408	-0.008**	-0.014, -0.001	-0.061**	-0.101, -0.021
Togo	Crude	0.268**	0.247 to 0.289	-0.013**	-0.016, -0.010	NA	NA
Togo	Standardized	0.247**	0.229 to 0.265	-0.012**	-0.015, -0.009	NA	NA
Togo	Standardized (adjusted)	0.250**	0.232 to 0.268	-0.010**	-0.014, -0.007	-0.071**	-0.086, -0.056
Turkey	Crude	0.106**	0.085 to 0.128	-0.003	-0.007 to 0.001	NA	NA
Turkey	Standardized	0.101**	0.076 to 0.125	-0.003	-0.006 to 0.001	NA	NA

		Constant		Mother's height		Household wealth	
Country	Estimate	Constant	95% CI	Coefficient	95% CI	Coefficient	95% CI
Turkey	Standardized (adjusted)	0.101**	0.076 to 0.125	-0.003	-0.006 to 0.001	-0.001	-0.020 to 0.017
Uganda	Crude	0.250**	0.234 to 0.267	-0.015**	-0.018, -0.013	NA	NA
Uganda	Standardized	0.225**	0.211 to 0.239	-0.014**	-0.016, -0.012	NA	NA
Uganda	Standardized (adjusted)	0.224**	0.209 to 0.238	-0.015**	-0.017, -0.013	-0.054**	-0.068, -0.040
Uzbekistan	Crude	0.373**	0.335 to 0.410	-0.004	-0.010 to 0.003	NA	NA
Uzbekistan	Standardized	0.363**	0.325 to 0.400	-0.004	-0.011 to 0.002	NA	NA
Uzbekistan	Standardized (adjusted)	0.363**	0.326 to 0.401	-0.004	-0.010 to 0.002	-0.037*	-0.079 to 0.005
Yemen	Crude	0.459**	0.443 to 0.476	-0.016**	-0.018, -0.014	NA	NA
Yemen	Standardized	0.360**	0.333 to 0.388	-0.011**	-0.016, -0.005	NA	NA
Yemen	Standardized (adjusted)	0.374**	0.345 to 0.403	-0.009**	-0.015, -0.003	-0.113**	-0.139, -0.086
Zambia	Crude	0.400**	0.388 to 0.412	-0.014**	-0.016, -0.012	NA	NA
Zambia	Standardized	0.358**	0.345 to 0.371	-0.012**	-0.015, -0.010	NA	NA
Zambia	Standardized (adjusted)	0.363**	0.350 to 0.377	-0.011**	-0.013, -0.008	-0.049**	-0.063, -0.034
Zimbabwe	Crude	0.236**	0.221 to 0.251	-0.013**	-0.016, -0.011	NA	NA
Zimbabwe	Standardized	0.234**	0.219 to 0.249	-0.013**	-0.016, -0.010	NA	NA
Zimbabwe	Standardized (adjusted)	0.234**	0.219 to 0.249	-0.012**	-0.015, -0.009	-0.038**	-0.052, -0.024

CI, confidence interval.

Notes: *p < .1; ** p < .05; *** p < .01. Each row shows parameters from as single ordinary least squares model with a binary indicator for stunting as an outcome. The constant shows the proportion stunted. All estimates were weighted using sampling weights which sum up to one in each survey. Standardized estimates were further re-scaled to sum up to the probability-density within each stratum of maternal height in the reference population. Maternal height was centered around mean maternal height in the reference population for Standardized models, and its sample mean for Crude models. Household wealth was centered around its sample mean. Standard errors were adjusted for clustering at the PSU-level for Crude estimates, and PSU crossed with maternal height for Standardized estimates.

eTable 5. Samples, survey years, and missing observations: male sub-sample

		Missing				
	Year	Stunted	Maternal height	Paternal height	Full sample	Analytical sample [%]
All	2013.3	20,336	3,986	250,200	250,200	228,802 [91.4]
Albania	2017–2018	182	23	1,708	1,708	1,519 [88.9]
Azerbaijan	2006	139	43	1,601	1,601	1,451 [90.6]
Dominican Republic	2013	285	22	1,472	1,472	1,177 [80.0]
Ethiopia	2016	475	97	2,451	2,451	1,947 [79.4]
Ghana	2014	113	6	998	998	883 [88.5]
Guyana	2009	191	34	680	680	480 [70.6]
India	2015–2016	13,295	2,700	213,168	213,168	199,133 [93.4]
Lesotho	2014	151	8	1,040	1,040	888 [85.4]
Liberia	2013	351	15	1,698	1,698	1,337 [78.7]
Maldives	2016–2017	295	104	1,441	1,441	1,137 [78.9]
Namibia	2013	543	81	1,655	1,655	1,085 [65.6]
Nepal	2016	64	7	1,164	1,164	1,095 [94.1]
Rwanda	2014–2015	350	174	3,915	3,915	3,559 [90.9]
Sao Tome & Principe	2008–2009	124	67	646	646	486 [75.2]
Senegal	2010–2011	439	141	1,992	1,992	1,515 [76.1]
Sierra Leone	2013	604	64	2,100	2,100	1,474 [70.2]
South Africa	2016	449	214	1,220	1,220	721 [59.1]
Swaziland	2006–2007	410	38	1,835	1,835	1,414 [77.1]
Timor-Leste	2016	793	57	4,969	4,969	4,152 [83.6]
Uganda	2016	425	26	1,896	1,896	1,468 [77.4]
Zimbabwe	2015	658	65	2,551	2,551	1,881 [73.7]

Notes: Percentages of full samples are shown in brackets.

eTable 6. Result of father's height measurements

	Observations (%)
Measured	62,426 (87.0%)
Implausible values	679 (0.9%)
Not present	3,759 (5.2%)
Refused	1,409 (2.0%)
Unknown reason	3,507 (4.9%)
Total	71,780

Notes: Height-for-age z-scores (HAZ) above 6 or below -6 were grouped as implausible. No measure found in household (HH) usually occurs when households were not sampled for anthropometric measures but in these cases, it was not possible to establish with full certainty. Children in households not selected for height measurement were excluded.

eTable 7. Average maternal and paternal height: male sub-sample

Country	Mother's height		Father's height		Obs.
	Mean [SD]	95% CI	Mean [SD]	95% CI	
All	157.46 [6.9]	(157.27 to 157.6)	168.93 [7.5]	(168.73 to 169.1)	58,933
Albania	160.53 [6.8]	(159.71 to 161.3)	173.42 [6.8]	(172.63 to 174.2)	842
Azerbaijan	158.30 [5.3]	(157.53 to 159.1)	172.33 [5.5]	(171.49 to 173.2)	475
Dominican Republic	158.73 [6.2]	(158.27 to 159.2)	171.01 [6.7]	(170.46 to 171.6)	1,705
Ethiopia	157.10 [6.0]	(156.79 to 157.4)	168.60 [6.6]	(168.22 to 169.0)	5,613
Ghana	159.15 [5.7]	(158.76 to 159.5)	170.36 [6.5]	(169.80 to 170.9)	1,631
Guyana	155.78 [7.4]	(154.61 to 156.9)	167.27 [8.2]	(165.89 to 168.6)	797
India	151.84 [5.8]	(151.72 to 152.0)	163.65 [6.8]	(163.50 to 163.8)	29,998
Lesotho	157.18 [5.4]	(156.56 to 157.8)	168.27 [6.4]	(167.48 to 169.1)	408
Liberia	157.11 [6.1]	(156.57 to 157.7)	166.98 [6.7]	(166.31 to 167.6)	1,714
Maldives	152.35 [5.2]	(151.83 to 152.9)	164.78 [6.3]	(164.11 to 165.4)	907
Namibia	161.22 [6.1]	(160.62 to 161.8)	171.84 [6.8]	(171.13 to 172.6)	596
Nepal	151.13 [5.4]	(150.67 to 151.6)	163.47 [6.2]	(162.95 to 164.0)	1,205
Sao Tome & Principe	159.45 [7.2]	(158.55 to 160.3)	170.97 [7.1]	(169.95 to 172.0)	722
Senegal	163.41 [6.2]	(162.95 to 163.9)	174.96 [7.0]	(174.36 to 175.5)	1,864
Sierra Leone	157.30 [6.3]	(156.89 to 157.7)	166.99 [6.6]	(166.55 to 167.4)	2,642
South Africa	158.24 [5.5]	(157.17 to 159.3)	170.52 [6.3]	(169.49 to 171.6)	234
Swaziland	159.04 [6.0]	(158.35 to 159.7)	170.37 [6.0]	(169.78 to 171.0)	545
Timor-Leste	151.92 [5.5]	(151.50 to 152.3)	161.73 [5.9]	(161.27 to 162.2)	1,667
Uganda	158.95 [6.3]	(158.55 to 159.3)	169.38 [6.9]	(168.96 to 169.8)	2,724
Zimbabwe	160.38 [6.1]	(160.03 to 160.7)	171.65 [6.6]	(171.30 to 172.0)	2,644

CI, confidence interval; SD, standard deviation.

Notes: All estimates were weighted using sampling weights which sum up to one for each survey. Standard errors were adjusted for clustering at the PSU-level.

eFigure 10. Parental height-standardized prevalence of stunting: male sub-sample

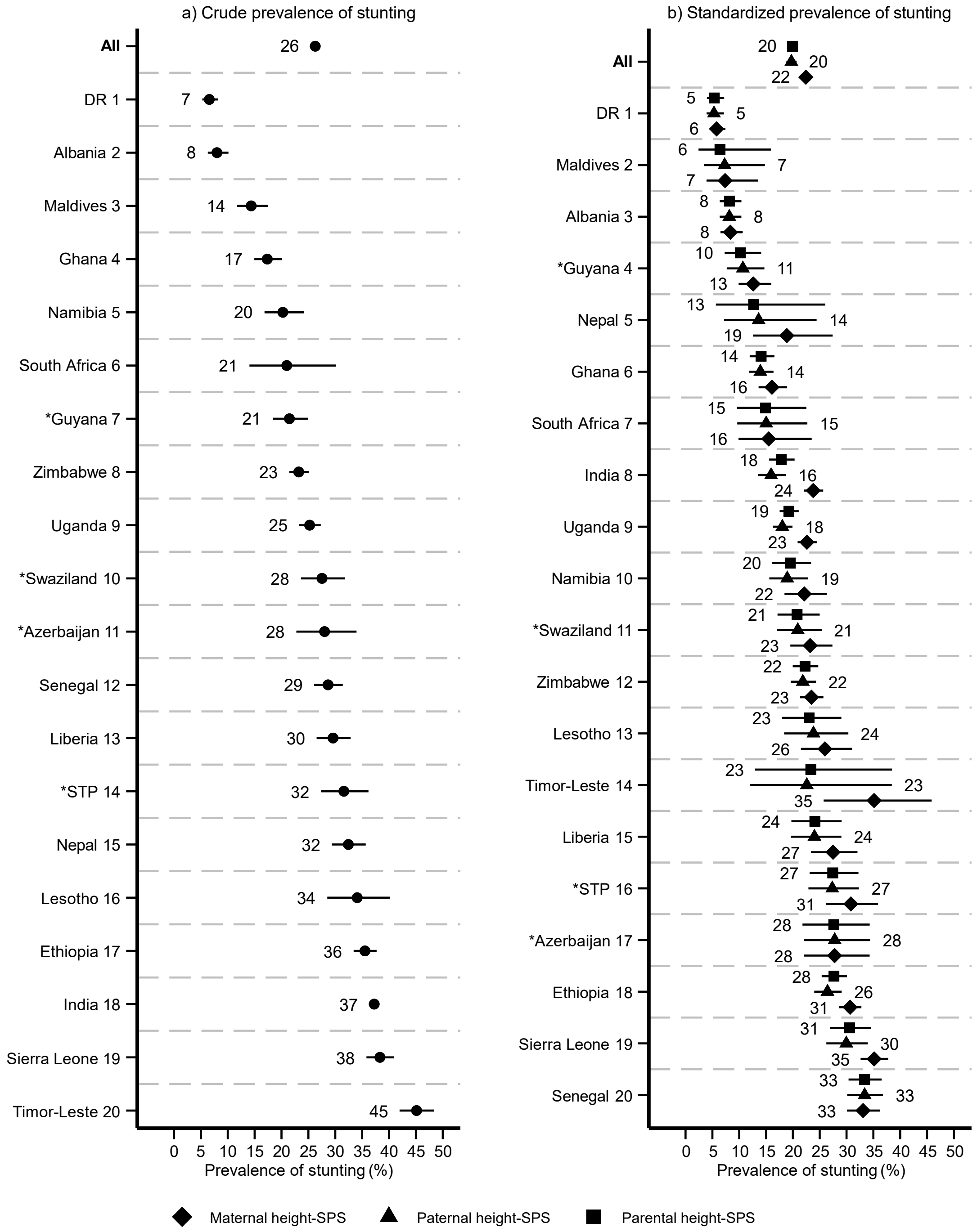

Notes: *Indicates estimates from surveys conducted before 2010. All estimates were weighted using sampling weights which sum up to one for each survey. Standard errors were adjusted for clustering at the PSU-level for CPS and PSU crossed with parental height for SPS. 95% confidence intervals are shown. Sao Tome and Principe (STP), Dominican Republic (DR).

eFigure 11. Mid-parental height-standardized prevalence of stunting: male sub-sample

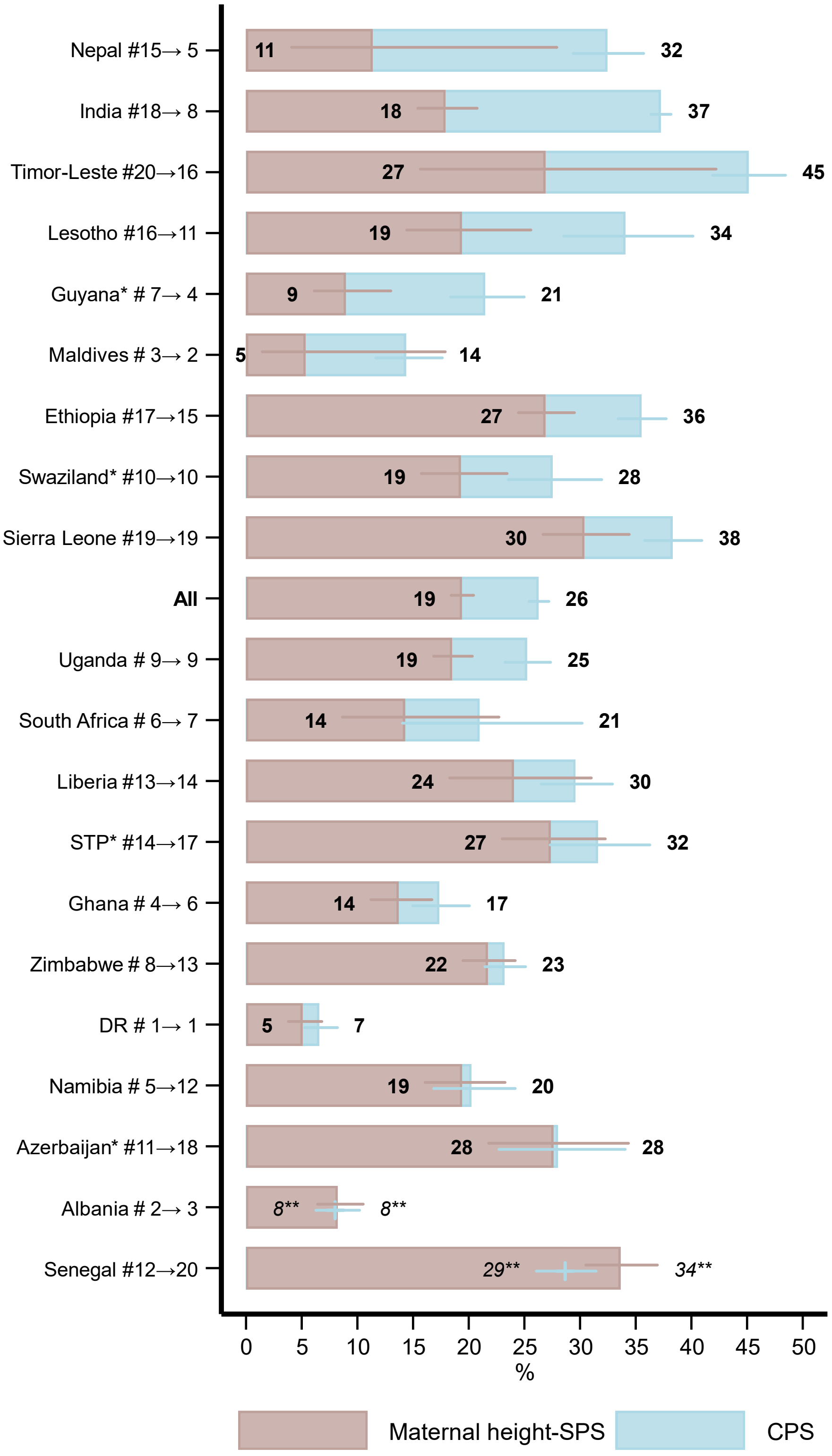

Notes: *Indicates estimates from surveys conducted before 2010. All estimates are weighted using sampling weights. Standard errors were adjusted for clustering at the PSU-level for CPS and PSU crossed with mid-parental height-level for SPS. 95% confidence intervals are shown. Sao Tome and Principe (STP), Dominican Republic (DR).

eTable 8. Results from OLS regressions estimating crude, standardized, and standardized and adjusted prevalence of stunting as well as regression parameters: male sub-sample

			Constant		Mother's height		Father's height		Household wealth		Mid-parental height	
Country	Estimate	Parent	Constant	95% CI	Coefficient	95% CI	Coefficient	95% CI	Coefficient	95% CI	Coefficient	95% CI
All	Crude	Mother	0.263**	0.254 to 0.272	-0.012**	-0.013, -0.011	NA	NA	NA	NA	NA	NA
All	Standardized	Mother	0.224**	0.215 to 0.234	-0.011**	-0.012, -0.009	NA	NA	NA	NA	NA	NA
All	Standardized (adjusted)	Mother	0.230**	0.221 to 0.240	-0.009**	-0.010, -0.007	-0.005**	-0.007, -0.004	-0.049**	-0.058, -0.040	NA	NA
All	Crude	Father	0.263**	0.254 to 0.272	NA	NA	-0.010**	-0.011, -0.009	NA	NA	NA	NA
All	Standardized	Father	0.197**	0.188 to 0.207	NA	NA	-0.008**	-0.009, -0.006	NA	NA	NA	NA
All	Standardized (adjusted)	Father	0.235**	0.225 to 0.246	-0.009**	-0.010, -0.007	-0.005**	-0.007, -0.004	-0.049**	-0.058, -0.040	NA	NA
All	Crude	Both	0.263**	0.254 to 0.272	-0.009**	-0.011, -0.008	-0.007**	-0.008, -0.006	NA	NA	NA	NA
All	Standardized	Both	0.200**	0.190 to 0.209	-0.009**	-0.011, -0.008	-0.006**	-0.007, -0.005	NA	NA	NA	NA
All	Standardized (adjusted)	Both	0.204**	0.195 to 0.214	-0.009**	-0.010, -0.007	-0.005**	-0.007, -0.004	-0.049**	-0.058, -0.040	NA	NA
All	Crude	Mid	0.263**	0.254 to 0.272	NA	NA	NA	NA	NA	NA	-0.016**	-0.017, -0.015
All	Standardized	Mid	0.194**	0.184 to 0.204	NA	NA	NA	NA	NA	NA	-0.015**	-0.017, -0.014
All	Standardized (adjusted)	Mid	0.198**	0.188 to 0.208	NA	NA	NA	NA	-0.049**	-0.058, -0.040	-0.014**	-0.015, -0.013
Albania	Crude	Mother	0.080**	0.060 to 0.100	-0.002	-0.005 to 0.002	NA	NA	NA	NA	NA	NA
Albania	Standardized	Mother	0.084**	0.063 to 0.104	-0.002	-0.006 to 0.001	NA	NA	NA	NA	NA	NA
Albania	Standardized (adjusted)	Mother	0.081**	0.061 to 0.100	0.000	-0.003 to 0.004	-0.003	-0.006 to 0.001	-0.055**	-0.077, -0.032	NA	NA
Albania	Crude	Father	0.080**	0.061 to 0.099	NA	NA	-0.004**	-0.007, -0.001	NA	NA	NA	NA
Albania	Standardized	Father	0.082**	0.062 to 0.102	NA	NA	-0.004**	-0.008, -0.001	NA	NA	NA	NA
Albania	Standardized (adjusted)	Father	0.079**	0.060 to 0.099	0.000	-0.003 to 0.004	-0.003	-0.006 to 0.001	-0.055**	-0.077, -0.032	NA	NA
Albania	Crude	Both	0.080**	0.061 to 0.099	-0.000	-0.003 to 0.003	-0.004**	-0.007, -0.001	NA	NA	NA	NA
Albania	Standardized	Both	0.082**	0.062 to 0.102	-0.001	-0.004 to 0.002	-0.004**	-0.007, -0.001	NA	NA	NA	NA
Albania	Standardized (adjusted)	Both	0.080**	0.061 to 0.099	0.000	-0.003 to 0.004	-0.003	-0.006 to 0.001	-0.055**	-0.077, -0.032	NA	NA
Albania	Crude	Mid	0.080**	0.061 to 0.099	NA	NA	NA	NA	NA	NA	-0.004**	-0.008, -0.000

			Constant		Mother's height		Father's height		Household wealth		Mid-parental height	
Country	Estimate	Parent	Constant	95% CI	Coefficient	95% CI	Coefficient	95% CI	Coefficient	95% CI	Coefficient	95% CI
Albania	Standardized	Mid	0.082**	0.062 to 0.102	NA	NA	NA	NA	NA	NA	-0.005**	-0.009, -0.001
Albania	Standardized (adjusted)	Mid	0.080**	0.061 to 0.100	NA	NA	NA	NA	-0.054**	-0.077, -0.032	-0.002	-0.006 to 0.002
Azerbaijan	Crude	Mother	0.280**	0.224 to 0.336	0.005	-0.004 to 0.014	NA	NA	NA	NA	NA	NA
Azerbaijan	Standardized	Mother	0.278**	0.216 to 0.339	-0.004	-0.014 to 0.007	NA	NA	NA	NA	NA	NA
Azerbaijan	Standardized (adjusted)	Mother	0.282**	0.222 to 0.342	-0.003	-0.013 to 0.007	-0.000	-0.010 to 0.009	-0.035	-0.103 to 0.032	NA	NA
Azerbaijan	Crude	Father	0.280**	0.223 to 0.337	NA	NA	0.000	-0.010 to 0.011	NA	NA	NA	NA
Azerbaijan	Standardized	Father	0.278**	0.216 to 0.340	NA	NA	-0.003	-0.014 to 0.008	NA	NA	NA	NA
Azerbaijan	Standardized (adjusted)	Father	0.289**	0.229 to 0.348	-0.003	-0.013 to 0.007	-0.000	-0.010 to 0.009	-0.035	-0.103 to 0.032	NA	NA
Azerbaijan	Crude	Both	0.280**	0.224 to 0.336	0.005	-0.004 to 0.014	-0.000	-0.010 to 0.010	NA	NA	NA	NA
Azerbaijan	Standardized	Both	0.276**	0.213 to 0.339	-0.003	-0.013 to 0.007	-0.002	-0.013 to 0.008	NA	NA	NA	NA
Azerbaijan	Standardized (adjusted)	Both	0.282**	0.219 to 0.344	-0.003	-0.013 to 0.007	-0.000	-0.010 to 0.009	-0.035	-0.103 to 0.032	NA	NA
Azerbaijan	Crude	Mid	0.280**	0.224 to 0.337	NA	NA	NA	NA	NA	NA	0.005	-0.009 to 0.019
Azerbaijan	Standardized	Mid	0.276**	0.213 to 0.339	NA	NA	NA	NA	NA	NA	-0.005	-0.019 to 0.008
Azerbaijan	Standardized (adjusted)	Mid	0.281**	0.219 to 0.344	NA	NA	NA	NA	-0.034	-0.104 to 0.035	-0.003	-0.017 to 0.010
Dominican Republic	Crude	Mother	0.066**	0.051 to 0.080	-0.004**	-0.007, -0.002	NA	NA	NA	NA	NA	NA
Dominican Republic	Standardized	Mother	0.058**	0.043 to 0.073	-0.005**	-0.007, -0.002	NA	NA	NA	NA	NA	NA
Dominican Republic	Standardized (adjusted)	Mother	0.058**	0.044 to 0.073	-0.004**	-0.007, -0.002	-0.001	-0.004 to 0.002	-0.024**	-0.039, -0.010	NA	NA
Dominican Republic	Crude	Father	0.066**	0.051 to 0.080	NA	NA	-0.003	-0.006 to 0.001	NA	NA	NA	NA
Dominican Republic	Standardized	Father	0.053**	0.037 to 0.069	NA	NA	-0.002	-0.005 to 0.001	NA	NA	NA	NA
Dominican Republic	Standardized (adjusted)	Father	0.065**	0.047 to 0.083	-0.004**	-0.007, -0.002	-0.001	-0.004 to 0.002	-0.024**	-0.039, -0.010	NA	NA
Dominican Republic	Crude	Both	0.066**	0.051 to 0.080	-0.004**	-0.007, -0.002	-0.002	-0.006 to 0.001	NA	NA	NA	NA
Dominican Republic	Standardized	Both	0.054**	0.038 to 0.070	-0.004**	-0.007, -0.002	-0.002	-0.005 to 0.001	NA	NA	NA	NA
Dominican Republic	Standardized (adjusted)	Both	0.055**	0.039 to 0.071	-0.004**	-0.007, -0.002	-0.001	-0.004 to 0.002	-0.024**	-0.039, -0.010	NA	NA
Dominican Republic	Crude	Mid	0.066**	0.051 to 0.080	NA	NA	NA	NA	NA	NA	-0.006**	-0.011, -0.002

			Constant		Mother's height		Father's height		Household wealth		Mid-parental height	
Country	Estimate	Parent	Constant	95% CI	Coefficient	95% CI	Coefficient	95% CI	Coefficient	95% CI	Coefficient	95% CI
Dominican Republic	Standardized	Mid	0.051**	0.036 to 0.065	NA	NA	NA	NA	NA	NA	-0.006**	-0.010, -0.002
Dominican Republic	Standardized (adjusted)	Mid	0.052**	0.037 to 0.066	NA	NA	NA	NA	-0.022**	-0.037, -0.008	-0.005**	-0.009, -0.002
Ethiopia	Crude	Mother	0.355**	0.334 to 0.377	-0.014**	-0.017, -0.011	NA	NA	NA	NA	NA	NA
Ethiopia	Standardized	Mother	0.307**	0.286 to 0.327	-0.012**	-0.016, -0.009	NA	NA	NA	NA	NA	NA
Ethiopia	Standardized (adjusted)	Mother	0.315**	0.294 to 0.336	-0.010**	-0.014, -0.007	-0.007**	-0.010, -0.004	-0.077**	-0.095, -0.060	NA	NA
Ethiopia	Crude	Father	0.355**	0.334 to 0.377	NA	NA	-0.010**	-0.013, -0.007	NA	NA	NA	NA
Ethiopia	Standardized	Father	0.265**	0.239 to 0.290	NA	NA	-0.010**	-0.013, -0.007	NA	NA	NA	NA
Ethiopia	Standardized (adjusted)	Father	0.318**	0.295 to 0.341	-0.010**	-0.014, -0.007	-0.007**	-0.010, -0.004	-0.077**	-0.095, -0.060	NA	NA
Ethiopia	Crude	Both	0.355**	0.334 to 0.377	-0.013**	-0.016, -0.010	-0.008**	-0.011, -0.005	NA	NA	NA	NA
Ethiopia	Standardized	Both	0.277**	0.253 to 0.300	-0.011**	-0.014, -0.007	-0.007**	-0.010, -0.004	NA	NA	NA	NA
Ethiopia	Standardized (adjusted)	Both	0.278**	0.255 to 0.302	-0.010**	-0.014, -0.007	-0.007**	-0.010, -0.004	-0.077**	-0.095, -0.060	NA	NA
Ethiopia	Crude	Mid	0.355**	0.334 to 0.377	NA	NA	NA	NA	NA	NA	-0.020**	-0.024, -0.017
Ethiopia	Standardized	Mid	0.269**	0.244 to 0.294	NA	NA	NA	NA	NA	NA	-0.018**	-0.022, -0.014
Ethiopia	Standardized (adjusted)	Mid	0.271**	0.246 to 0.296	NA	NA	NA	NA	-0.078**	-0.095, -0.061	-0.017**	-0.022, -0.013
Ghana	Crude	Mother	0.174**	0.147 to 0.200	-0.013**	-0.018, -0.008	NA	NA	NA	NA	NA	NA
Ghana	Standardized	Mother	0.161**	0.134 to 0.188	-0.009**	-0.015, -0.004	NA	NA	NA	NA	NA	NA
Ghana	Standardized (adjusted)	Mother	0.161**	0.136 to 0.185	-0.009**	-0.014, -0.004	-0.005**	-0.009, -0.002	-0.062**	-0.085, -0.040	NA	NA
Ghana	Crude	Father	0.174**	0.147 to 0.200	NA	NA	-0.007**	-0.010, -0.004	NA	NA	NA	NA
Ghana	Standardized	Father	0.139**	0.117 to 0.162	NA	NA	-0.007**	-0.011, -0.004	NA	NA	NA	NA
Ghana	Standardized (adjusted)	Father	0.158**	0.136 to 0.180	-0.009**	-0.014, -0.004	-0.005**	-0.009, -0.002	-0.062**	-0.085, -0.040	NA	NA
Ghana	Crude	Both	0.174**	0.148 to 0.199	-0.012**	-0.018, -0.007	-0.005**	-0.008, -0.003	NA	NA	NA	NA
Ghana	Standardized	Both	0.141**	0.118 to 0.164	-0.009**	-0.014, -0.003	-0.006**	-0.009, -0.003	NA	NA	NA	NA
Ghana	Standardized (adjusted)	Both	0.142**	0.121 to 0.164	-0.009**	-0.014, -0.004	-0.005**	-0.009, -0.002	-0.062**	-0.085, -0.040	NA	NA
Ghana	Crude	Mid	0.174**	0.148 to 0.199	NA	NA	NA	NA	NA	NA	-0.017**	-0.023, -0.010

			Constant		Mother's height		Father's height		Household wealth		Mid-parental height	
Country	Estimate	Parent	Constant	95% CI	Coefficient	95% CI	Coefficient	95% CI	Coefficient	95% CI	Coefficient	95% CI
Ghana	Standardized	Mid	0.137**	0.110 to 0.164	NA	NA	NA	NA	NA	NA	-0.015**	-0.021, -0.009
Ghana	Standardized (adjusted)	Mid	0.137**	0.113 to 0.161	NA	NA	NA	NA	-0.062**	-0.084, -0.039	-0.014**	-0.020, -0.008
Guyana	Crude	Mother	0.215**	0.179 to 0.250	-0.013**	-0.020, -0.007	NA	NA	NA	NA	NA	NA
Guyana	Standardized	Mother	0.126**	0.096 to 0.156	-0.013**	-0.017, -0.009	NA	NA	NA	NA	NA	NA
Guyana	Standardized (adjusted)	Mother	0.133**	0.103 to 0.163	-0.012**	-0.016, -0.007	-0.004**	-0.008, -0.001	-0.008	-0.043 to 0.026	NA	NA
Guyana	Crude	Father	0.215**	0.179 to 0.251	NA	NA	-0.013**	-0.018, -0.008	NA	NA	NA	NA
Guyana	Standardized	Father	0.107**	0.072 to 0.142	NA	NA	-0.005**	-0.010, -0.001	NA	NA	NA	NA
Guyana	Standardized (adjusted)	Father	0.166**	0.128 to 0.204	-0.012**	-0.016, -0.007	-0.004**	-0.008, -0.001	-0.008	-0.043 to 0.026	NA	NA
Guyana	Crude	Both	0.215**	0.182 to 0.248	-0.009**	-0.016, -0.003	-0.010**	-0.014, -0.006	NA	NA	NA	NA
Guyana	Standardized	Both	0.102**	0.068 to 0.136	-0.012**	-0.016, -0.008	-0.004**	-0.008, -0.001	NA	NA	NA	NA
Guyana	Standardized (adjusted)	Both	0.106**	0.074 to 0.137	-0.012**	-0.016, -0.007	-0.004**	-0.008, -0.001	-0.008	-0.043 to 0.026	NA	NA
Guyana	Crude	Mid	0.215**	0.182 to 0.248	NA	NA	NA	NA	NA	NA	-0.019**	-0.026, -0.013
Guyana	Standardized	Mid	0.090**	0.056 to 0.123	NA	NA	NA	NA	NA	NA	-0.015**	-0.020, -0.010
Guyana	Standardized (adjusted)	Mid	0.096**	0.065 to 0.128	NA	NA	NA	NA	-0.014	-0.048 to 0.021	-0.014**	-0.019, -0.008
India	Crude	Mother	0.372**	0.364 to 0.381	-0.017**	-0.019, -0.016	NA	NA	NA	NA	NA	NA
India	Standardized	Mother	0.238**	0.220 to 0.256	-0.011**	-0.015, -0.007	NA	NA	NA	NA	NA	NA
India	Standardized (adjusted)	Mother	0.278**	0.254 to 0.302	-0.007**	-0.011, -0.003	-0.006**	-0.009, -0.003	-0.069**	-0.086, -0.051	NA	NA
India	Crude	Father	0.372**	0.364 to 0.381	NA	NA	-0.012**	-0.013, -0.011	NA	NA	NA	NA
India	Standardized	Father	0.159**	0.134 to 0.185	NA	NA	-0.011**	-0.014, -0.009	NA	NA	NA	NA
India	Standardized (adjusted)	Father	0.284**	0.242 to 0.326	-0.007**	-0.011, -0.003	-0.006**	-0.009, -0.003	-0.069**	-0.086, -0.051	NA	NA
India	Crude	Both	0.372**	0.364 to 0.381	-0.015**	-0.016, -0.014	-0.009**	-0.010, -0.008	NA	NA	NA	NA
India	Standardized	Both	0.178**	0.155 to 0.202	-0.008**	-0.012, -0.004	-0.008**	-0.012, -0.005	NA	NA	NA	NA
India	Standardized (adjusted)	Both	0.220**	0.193 to 0.246	-0.007**	-0.011, -0.003	-0.006**	-0.009, -0.003	-0.069**	-0.086, -0.051	NA	NA
India	Crude	Mid	0.372**	0.364 to 0.381	NA	NA	NA	NA	NA	NA	-0.023**	-0.025, -0.022

			Constant		Mother's height		Father's height		Household wealth		Mid-parental height	
Country	Estimate	Parent	Constant	95% CI	Coefficient	95% CI	Coefficient	95% CI	Coefficient	95% CI	Coefficient	95% CI
India	Standardized	Mid	0.179**	0.153 to 0.206	NA	NA	NA	NA	NA	NA	-0.016**	-0.020, -0.013
India	Standardized (adjusted)	Mid	0.215**	0.183 to 0.247	NA	NA	NA	NA	-0.068**	-0.087, -0.049	-0.013**	-0.016, -0.009
Lesotho	Crude	Mother	0.341**	0.282 to 0.399	-0.020**	-0.028, -0.012	NA	NA	NA	NA	NA	NA
Lesotho	Standardized	Mother	0.260**	0.212 to 0.308	-0.023**	-0.029, -0.016	NA	NA	NA	NA	NA	NA
Lesotho	Standardized (adjusted)	Mother	0.265**	0.217 to 0.313	-0.021**	-0.028, -0.015	-0.005	-0.012 to 0.001	-0.054**	-0.098, -0.009	NA	NA
Lesotho	Crude	Father	0.341**	0.283 to 0.399	NA	NA	-0.007*	-0.014 to 0.001	NA	NA	NA	NA
Lesotho	Standardized	Father	0.238**	0.178 to 0.298	NA	NA	-0.007**	-0.014, -0.000	NA	NA	NA	NA
Lesotho	Standardized (adjusted)	Father	0.317**	0.259 to 0.376	-0.021**	-0.028, -0.015	-0.005	-0.012 to 0.001	-0.054**	-0.098, -0.009	NA	NA
Lesotho	Crude	Both	0.341**	0.283 to 0.399	-0.019**	-0.027, -0.012	-0.006	-0.014 to 0.001	NA	NA	NA	NA
Lesotho	Standardized	Both	0.230**	0.175 to 0.286	-0.022**	-0.029, -0.016	-0.005	-0.012 to 0.001	NA	NA	NA	NA
Lesotho	Standardized (adjusted)	Both	0.237**	0.181 to 0.293	-0.021**	-0.028, -0.015	-0.005	-0.012 to 0.001	-0.054**	-0.098, -0.009	NA	NA
Lesotho	Crude	Mid	0.341**	0.283 to 0.399	NA	NA	NA	NA	NA	NA	-0.024**	-0.034, -0.013
Lesotho	Standardized	Mid	0.194**	0.138 to 0.249	NA	NA	NA	NA	NA	NA	-0.027**	-0.036, -0.018
Lesotho	Standardized (adjusted)	Mid	0.203**	0.145 to 0.260	NA	NA	NA	NA	-0.058**	-0.104, -0.011	-0.025**	-0.035, -0.016
Liberia	Crude	Mother	0.296**	0.264 to 0.328	-0.010**	-0.014, -0.006	NA	NA	NA	NA	NA	NA
Liberia	Standardized	Mother	0.275**	0.231 to 0.318	-0.005	-0.013 to 0.002	NA	NA	NA	NA	NA	NA
Liberia	Standardized (adjusted)	Mother	0.277**	0.234 to 0.320	-0.005	-0.013 to 0.003	-0.004**	-0.009, -0.000	-0.060**	-0.098, -0.023	NA	NA
Liberia	Crude	Father	0.296**	0.262 to 0.329	NA	NA	-0.007**	-0.010, -0.003	NA	NA	NA	NA
Liberia	Standardized	Father	0.240**	0.193 to 0.287	NA	NA	-0.005**	-0.010, -0.001	NA	NA	NA	NA
Liberia	Standardized (adjusted)	Father	0.265**	0.226 to 0.305	-0.005	-0.013 to 0.003	-0.004**	-0.009, -0.000	-0.060**	-0.098, -0.023	NA	NA
Liberia	Crude	Both	0.296**	0.264 to 0.328	-0.009**	-0.014, -0.005	-0.006**	-0.010, -0.002	NA	NA	NA	NA
Liberia	Standardized	Both	0.241**	0.194 to 0.288	-0.005	-0.013 to 0.003	-0.005**	-0.009, -0.001	NA	NA	NA	NA
Liberia	Standardized (adjusted)	Both	0.247**	0.201 to 0.293	-0.005	-0.013 to 0.003	-0.004**	-0.009, -0.000	-0.060**	-0.098, -0.023	NA	NA
Liberia	Crude	Mid	0.296**	0.264 to 0.328	NA	NA	NA	NA	NA	NA	-0.015**	-0.021, -0.009

			Constant		Mother's height		Father's height		Household wealth		Mid-parental height	
Country	Estimate	Parent	Constant	95% CI	Coefficient	95% CI	Coefficient	95% CI	Coefficient	95% CI	Coefficient	95% CI
Liberia	Standardized	Mid	0.241**	0.177 to 0.304	NA	NA	NA	NA	NA	NA	-0.010**	-0.019, -0.002
Liberia	Standardized (adjusted)	Mid	0.245**	0.182 to 0.308	NA	NA	NA	NA	-0.060**	-0.097, -0.023	-0.009**	-0.018, -0.001
Maldives	Crude	Mother	0.144**	0.115 to 0.172	-0.016**	-0.022, -0.009	NA	NA	NA	NA	NA	NA
Maldives	Standardized	Mother	0.074**	0.028 to 0.120	-0.005	-0.012 to 0.001	NA	NA	NA	NA	NA	NA
Maldives	Standardized (adjusted)	Mother	0.070**	0.030 to 0.109	-0.006*	-0.012 to 0.000	-0.001	-0.005 to 0.003	-0.029	-0.075 to 0.016	NA	NA
Maldives	Crude	Father	0.144**	0.113 to 0.175	NA	NA	-0.005	-0.010 to 0.001	NA	NA	NA	NA
Maldives	Standardized	Father	0.073**	0.019 to 0.126	NA	NA	-0.002	-0.006 to 0.003	NA	NA	NA	NA
Maldives	Standardized (adjusted)	Father	0.112**	0.065 to 0.159	-0.006*	-0.012 to 0.000	-0.001	-0.005 to 0.003	-0.029	-0.075 to 0.016	NA	NA
Maldives	Crude	Both	0.144**	0.115 to 0.172	-0.015**	-0.022, -0.009	-0.003	-0.009 to 0.002	NA	NA	NA	NA
Maldives	Standardized	Both	0.064**	0.003 to 0.125	-0.005	-0.012 to 0.002	-0.001	-0.005 to 0.003	NA	NA	NA	NA
Maldives	Standardized (adjusted)	Both	0.060**	0.005 to 0.116	-0.006*	-0.012 to 0.000	-0.001	-0.005 to 0.003	-0.029	-0.075 to 0.016	NA	NA
Maldives	Crude	Mid	0.144**	0.114 to 0.173	NA	NA	NA	NA	NA	NA	-0.016**	-0.024, -0.009
Maldives	Standardized	Mid	0.053	-0.015 to 0.122	NA	NA	NA	NA	NA	NA	-0.006	-0.014 to 0.002
Maldives	Standardized (adjusted)	Mid	0.049	-0.012 to 0.109	NA	NA	NA	NA	-0.025	-0.073 to 0.023	-0.007*	-0.014 to 0.001
Namibia	Crude	Mother	0.202**	0.165 to 0.240	-0.015**	-0.021, -0.008	NA	NA	NA	NA	NA	NA
Namibia	Standardized	Mother	0.221**	0.182 to 0.261	-0.016**	-0.023, -0.009	NA	NA	NA	NA	NA	NA
Namibia	Standardized (adjusted)	Mother	0.218**	0.181 to 0.256	-0.012**	-0.019, -0.005	-0.011**	-0.017, -0.005	-0.055**	-0.089, -0.021	NA	NA
Namibia	Crude	Father	0.202**	0.166 to 0.239	NA	NA	-0.013**	-0.018, -0.008	NA	NA	NA	NA
Namibia	Standardized	Father	0.190**	0.153 to 0.226	NA	NA	-0.015**	-0.020, -0.010	NA	NA	NA	NA
Namibia	Standardized (adjusted)	Father	0.194**	0.158 to 0.229	-0.012**	-0.019, -0.005	-0.011**	-0.017, -0.005	-0.055**	-0.089, -0.021	NA	NA
Namibia	Crude	Both	0.202**	0.166 to 0.239	-0.012**	-0.018, -0.005	-0.011**	-0.016, -0.006	NA	NA	NA	NA
Namibia	Standardized	Both	0.195**	0.159 to 0.231	-0.013**	-0.020, -0.005	-0.012**	-0.018, -0.006	NA	NA	NA	NA
Namibia	Standardized (adjusted)	Both	0.197**	0.160 to 0.233	-0.012**	-0.019, -0.005	-0.011**	-0.017, -0.005	-0.055**	-0.089, -0.021	NA	NA
Namibia	Crude	Mid	0.202**	0.166 to 0.239	NA	NA	NA	NA	NA	NA	-0.022**	-0.029, -0.016

			Constant		Mother's height		Father's height		Household wealth		Mid-parental height	
Country	Estimate	Parent	Constant	95% CI	Coefficient	95% CI	Coefficient	95% CI	Coefficient	95% CI	Coefficient	95% CI
Namibia	Standardized	Mid	0.194**	0.158 to 0.230	NA	NA	NA	NA	NA	NA	-0.024**	-0.031, -0.017
Namibia	Standardized (adjusted)	Mid	0.196**	0.160 to 0.231	NA	NA	NA	NA	-0.055**	-0.089, -0.021	-0.023**	-0.030, -0.016
Nepal	Crude	Mother	0.324**	0.293 to 0.356	-0.016**	-0.022, -0.010	NA	NA	NA	NA	NA	NA
Nepal	Standardized	Mother	0.189**	0.115 to 0.263	-0.013**	-0.023, -0.004	NA	NA	NA	NA	NA	NA
Nepal	Standardized (adjusted)	Mother	0.208**	0.132 to 0.284	-0.012**	-0.022, -0.002	-0.007**	-0.013, -0.000	-0.064**	-0.111, -0.016	NA	NA
Nepal	Crude	Father	0.324**	0.292 to 0.357	NA	NA	-0.015**	-0.019, -0.011	NA	NA	NA	NA
Nepal	Standardized	Father	0.136**	0.052 to 0.221	NA	NA	-0.009**	-0.017, -0.002	NA	NA	NA	NA
Nepal	Standardized (adjusted)	Father	0.260**	0.188 to 0.333	-0.012**	-0.022, -0.002	-0.007**	-0.013, -0.000	-0.064**	-0.111, -0.016	NA	NA
Nepal	Crude	Both	0.324**	0.293 to 0.356	-0.013**	-0.019, -0.007	-0.013**	-0.017, -0.009	NA	NA	NA	NA
Nepal	Standardized	Both	0.127**	0.029 to 0.225	-0.012**	-0.022, -0.002	-0.008**	-0.014, -0.001	NA	NA	NA	NA
Nepal	Standardized (adjusted)	Both	0.138**	0.038 to 0.238	-0.012**	-0.022, -0.002	-0.007**	-0.013, -0.000	-0.064**	-0.111, -0.016	NA	NA
Nepal	Crude	Mid	0.324**	0.293 to 0.356	NA	NA	NA	NA	NA	NA	-0.026**	-0.033, -0.020
Nepal	Standardized	Mid	0.114**	0.003 to 0.225	NA	NA	NA	NA	NA	NA	-0.019**	-0.031, -0.008
Nepal	Standardized (adjusted)	Mid	0.122**	0.010 to 0.234	NA	NA	NA	NA	-0.062**	-0.110, -0.014	-0.019**	-0.031, -0.007
Sao Tome & Principe	Crude	Mother	0.316**	0.270 to 0.362	-0.010**	-0.017, -0.004	NA	NA	NA	NA	NA	NA
Sao Tome & Principe	Standardized	Mother	0.308**	0.260 to 0.357	-0.013**	-0.020, -0.007	NA	NA	NA	NA	NA	NA
Sao Tome & Principe	Standardized (adjusted)	Mother	0.309**	0.263 to 0.356	-0.012**	-0.019, -0.005	-0.011**	-0.017, -0.004	-0.049**	-0.092, -0.006	NA	NA
Sao Tome & Principe	Crude	Father	0.316**	0.274 to 0.359	NA	NA	-0.013**	-0.019, -0.007	NA	NA	NA	NA
Sao Tome & Principe	Standardized	Father	0.273**	0.226 to 0.321	NA	NA	-0.013**	-0.019, -0.006	NA	NA	NA	NA
Sao Tome & Principe	Standardized (adjusted)	Father	0.298**	0.251 to 0.345	-0.012**	-0.019, -0.005	-0.011**	-0.017, -0.004	-0.049**	-0.092, -0.006	NA	NA
Sao Tome & Principe	Crude	Both	0.316**	0.272 to 0.360	-0.009**	-0.016, -0.003	-0.013**	-0.019, -0.007	NA	NA	NA	NA
Sao Tome & Principe	Standardized	Both	0.274**	0.229 to 0.320	-0.012**	-0.019, -0.006	-0.012**	-0.019, -0.005	NA	NA	NA	NA
Sao Tome & Principe	Standardized (adjusted)	Both	0.279**	0.233 to 0.325	-0.012**	-0.019, -0.005	-0.011**	-0.017, -0.004	-0.049**	-0.092, -0.006	NA	NA
Sao Tome & Principe	Crude	Mid	0.316**	0.271 to 0.361	NA	NA	NA	NA	NA	NA	-0.022**	-0.031, -0.013

			Constant		Mother's height		Father's height		Household wealth		Mid-parental height	
Country	Estimate	Parent	Constant	95% CI	Coefficient	95% CI	Coefficient	95% CI	Coefficient	95% CI	Coefficient	95% CI
Sao Tome & Principe	Standardized	Mid	0.274**	0.228 to 0.320	NA	NA	NA	NA	NA	NA	-0.024**	-0.032, -0.016
Sao Tome & Principe	Standardized (adjusted)	Mid	0.277**	0.230 to 0.324	NA	NA	NA	NA	-0.049**	-0.091, -0.006	-0.023**	-0.031, -0.014
Senegal	Crude	Mother	0.287**	0.259 to 0.314	-0.011**	-0.015, -0.006	NA	NA	NA	NA	NA	NA
Senegal	Standardized	Mother	0.331**	0.300 to 0.362	-0.015**	-0.021, -0.010	NA	NA	NA	NA	NA	NA
Senegal	Standardized (adjusted)	Mother	0.324**	0.292 to 0.356	-0.013**	-0.019, -0.008	-0.002	-0.008 to 0.003	-0.092**	-0.127, -0.058	NA	NA
Senegal	Crude	Father	0.287**	0.259 to 0.314	NA	NA	-0.006**	-0.012, -0.000	NA	NA	NA	NA
Senegal	Standardized	Father	0.334**	0.300 to 0.367	NA	NA	-0.007**	-0.013, -0.002	NA	NA	NA	NA
Senegal	Standardized (adjusted)	Father	0.295**	0.269 to 0.322	-0.013**	-0.019, -0.008	-0.002	-0.008 to 0.003	-0.092**	-0.127, -0.058	NA	NA
Senegal	Crude	Both	0.287**	0.260 to 0.313	-0.010**	-0.015, -0.005	-0.005	-0.011 to 0.001	NA	NA	NA	NA
Senegal	Standardized	Both	0.334**	0.303 to 0.365	-0.014**	-0.020, -0.009	-0.005*	-0.010 to 0.001	NA	NA	NA	NA
Senegal	Standardized (adjusted)	Both	0.327**	0.295 to 0.359	-0.013**	-0.019, -0.008	-0.002	-0.008 to 0.003	-0.092**	-0.127, -0.058	NA	NA
Senegal	Crude	Mid	0.287**	0.260 to 0.313	NA	NA	NA	NA	NA	NA	-0.014**	-0.021, -0.008
Senegal	Standardized	Mid	0.336**	0.304 to 0.368	NA	NA	NA	NA	NA	NA	-0.019**	-0.026, -0.011
Senegal	Standardized (adjusted)	Mid	0.330**	0.296 to 0.364	NA	NA	NA	NA	-0.089**	-0.125, -0.053	-0.015**	-0.023, -0.007
Sierra Leone	Crude	Mother	0.383**	0.357 to 0.409	-0.012**	-0.016, -0.009	NA	NA	NA	NA	NA	NA
Sierra Leone	Standardized	Mother	0.351**	0.326 to 0.377	-0.008**	-0.013, -0.004	NA	NA	NA	NA	NA	NA
Sierra Leone	Standardized (adjusted)	Mother	0.357**	0.331 to 0.382	-0.007**	-0.012, -0.002	-0.006**	-0.011, -0.002	-0.037**	-0.073, -0.001	NA	NA
Sierra Leone	Crude	Father	0.383**	0.357 to 0.410	NA	NA	-0.010**	-0.013, -0.006	NA	NA	NA	NA
Sierra Leone	Standardized	Father	0.300**	0.261 to 0.338	NA	NA	-0.008**	-0.012, -0.004	NA	NA	NA	NA
Sierra Leone	Standardized (adjusted)	Father	0.339**	0.304 to 0.375	-0.007**	-0.012, -0.002	-0.006**	-0.011, -0.002	-0.037**	-0.073, -0.001	NA	NA
Sierra Leone	Crude	Both	0.383**	0.358 to 0.409	-0.011**	-0.015, -0.008	-0.008**	-0.012, -0.005	NA	NA	NA	NA
Sierra Leone	Standardized	Both	0.306**	0.267 to 0.344	-0.008**	-0.012, -0.003	-0.007**	-0.011, -0.003	NA	NA	NA	NA
Sierra Leone	Standardized (adjusted)	Both	0.313**	0.276 to 0.350	-0.007**	-0.012, -0.002	-0.006**	-0.011, -0.002	-0.037**	-0.073, -0.001	NA	NA
Sierra Leone	Crude	Mid	0.383**	0.358 to 0.409	NA	NA	NA	NA	NA	NA	-0.019**	-0.024, -0.014

			Constant		Mother's height		Father's height		Household wealth		Mid-parental height	
Country	Estimate	Parent	Constant	95% CI	Coefficient	95% CI	Coefficient	95% CI	Coefficient	95% CI	Coefficient	95% CI
Sierra Leone	Standardized	Mid	0.304**	0.265 to 0.342	NA	NA	NA	NA	NA	NA	-0.015**	-0.021, -0.009
Sierra Leone	Standardized (adjusted)	Mid	0.311**	0.273 to 0.349	NA	NA	NA	NA	-0.037**	-0.073, -0.001	-0.013**	-0.019, -0.007
South Africa	Crude	Mother	0.210**	0.129 to 0.290	-0.009	-0.023 to 0.004	NA	NA	NA	NA	NA	NA
South Africa	Standardized	Mother	0.155**	0.087 to 0.223	-0.008*	-0.018 to 0.001	NA	NA	NA	NA	NA	NA
South Africa	Standardized (adjusted)	Mother	0.155**	0.087 to 0.223	-0.008	-0.018 to 0.002	-0.000	-0.008 to 0.007	-0.065**	-0.117, -0.012	NA	NA
South Africa	Crude	Father	0.210**	0.129 to 0.291	NA	NA	-0.005	-0.015 to 0.005	NA	NA	NA	NA
South Africa	Standardized	Father	0.150**	0.086 to 0.215	NA	NA	-0.004	-0.011 to 0.004	NA	NA	NA	NA
South Africa	Standardized (adjusted)	Father	0.176**	0.116 to 0.236	-0.008	-0.018 to 0.002	-0.000	-0.008 to 0.007	-0.065**	-0.117, -0.012	NA	NA
South Africa	Crude	Both	0.210**	0.129 to 0.290	-0.009	-0.023 to 0.005	-0.004	-0.014 to 0.007	NA	NA	NA	NA
South Africa	Standardized	Both	0.149**	0.085 to 0.213	-0.008	-0.018 to 0.002	-0.002	-0.010 to 0.006	NA	NA	NA	NA
South Africa	Standardized (adjusted)	Both	0.154**	0.088 to 0.219	-0.008	-0.018 to 0.002	-0.000	-0.008 to 0.007	-0.065**	-0.117, -0.012	NA	NA
South Africa	Crude	Mid	0.210**	0.129 to 0.290	NA	NA	NA	NA	NA	NA	-0.012*	-0.025 to 0.001
South Africa	Standardized	Mid	0.143**	0.074 to 0.212	NA	NA	NA	NA	NA	NA	-0.010*	-0.020 to 0.000
South Africa	Standardized (adjusted)	Mid	0.145**	0.074 to 0.216	NA	NA	NA	NA	-0.061**	-0.116, -0.006	-0.008	-0.019 to 0.002
Swaziland	Crude	Mother	0.275**	0.234 to 0.316	-0.021**	-0.027, -0.015	NA	NA	NA	NA	NA	NA
Swaziland	Standardized	Mother	0.232**	0.193 to 0.271	-0.017**	-0.023, -0.011	NA	NA	NA	NA	NA	NA
Swaziland	Standardized (adjusted)	Mother	0.235**	0.196 to 0.274	-0.016**	-0.022, -0.010	-0.007**	-0.014, -0.000	-0.057**	-0.091, -0.024	NA	NA
Swaziland	Crude	Father	0.275**	0.232 to 0.319	NA	NA	-0.009**	-0.016, -0.001	NA	NA	NA	NA
Swaziland	Standardized	Father	0.209**	0.168 to 0.251	NA	NA	-0.009**	-0.016, -0.002	NA	NA	NA	NA
Swaziland	Standardized (adjusted)	Father	0.242**	0.200 to 0.284	-0.016**	-0.022, -0.010	-0.007**	-0.014, -0.000	-0.057**	-0.091, -0.024	NA	NA
Swaziland	Crude	Both	0.275**	0.234 to 0.317	-0.020**	-0.026, -0.014	-0.007**	-0.014, -0.000	NA	NA	NA	NA
Swaziland	Standardized	Both	0.208**	0.169 to 0.247	-0.017**	-0.023, -0.011	-0.007**	-0.014, -0.001	NA	NA	NA	NA
Swaziland	Standardized (adjusted)	Both	0.211**	0.172 to 0.251	-0.016**	-0.022, -0.010	-0.007**	-0.014, -0.000	-0.057**	-0.091, -0.024	NA	NA
Swaziland	Crude	Mid	0.275**	0.234 to 0.317	NA	NA	NA	NA	NA	NA	-0.027**	-0.036, -0.019

			Constant		Mother's height		Father's height		Household wealth		Mid-parental height	
Country	Estimate	Parent	Constant	95% CI	Coefficient	95% CI	Coefficient	95% CI	Coefficient	95% CI	Coefficient	95% CI
Swaziland	Standardized	Mid	0.193**	0.155 to 0.231	NA	NA	NA	NA	NA	NA	-0.025**	-0.033, -0.017
Swaziland	Standardized (adjusted)	Mid	0.198**	0.159 to 0.236	NA	NA	NA	NA	-0.059**	-0.092, -0.026	-0.023**	-0.032, -0.015
Timor-Leste	Crude	Mother	0.451**	0.419 to 0.483	-0.017**	-0.022, -0.012	NA	NA	NA	NA	NA	NA
Timor-Leste	Standardized	Mother	0.351**	0.249 to 0.453	-0.008	-0.023 to 0.007	NA	NA	NA	NA	NA	NA
Timor-Leste	Standardized (adjusted)	Mother	0.384**	0.271 to 0.496	-0.004	-0.020 to 0.012	-0.012*	-0.027 to 0.002	-0.002	-0.072 to 0.067	NA	NA
Timor-Leste	Crude	Father	0.451**	0.419 to 0.484	NA	NA	-0.003	-0.008 to 0.002	NA	NA	NA	NA
Timor-Leste	Standardized	Father	0.226**	0.093 to 0.359	NA	NA	-0.014**	-0.025, -0.003	NA	NA	NA	NA
Timor-Leste	Standardized (adjusted)	Father	0.270**	0.063 to 0.476	-0.004	-0.020 to 0.012	-0.012*	-0.027 to 0.002	-0.002	-0.072 to 0.067	NA	NA
Timor-Leste	Crude	Both	0.451**	0.419 to 0.483	-0.017**	-0.022, -0.012	0.000	-0.004 to 0.005	NA	NA	NA	NA
Timor-Leste	Standardized	Both	0.233**	0.105 to 0.362	-0.004	-0.020 to 0.012	-0.012*	-0.026 to 0.001	NA	NA	NA	NA
Timor-Leste	Standardized (adjusted)	Both	0.234**	0.097 to 0.372	-0.004	-0.020 to 0.012	-0.012*	-0.027 to 0.002	-0.002	-0.072 to 0.067	NA	NA
Timor-Leste	Crude	Mid	0.451**	0.419 to 0.484	NA	NA	NA	NA	NA	NA	-0.015**	-0.022, -0.009
Timor-Leste	Standardized	Mid	0.269**	0.134 to 0.404	NA	NA	NA	NA	NA	NA	-0.017**	-0.029, -0.004
Timor-Leste	Standardized (adjusted)	Mid	0.270**	0.133 to 0.408	NA	NA	NA	NA	-0.007	-0.072 to 0.059	-0.016**	-0.029, -0.004
Uganda	Crude	Mother	0.252**	0.232 to 0.273	-0.016**	-0.019, -0.013	NA	NA	NA	NA	NA	NA
Uganda	Standardized	Mother	0.226**	0.208 to 0.244	-0.015**	-0.018, -0.012	NA	NA	NA	NA	NA	NA
Uganda	Standardized (adjusted)	Mother	0.230**	0.212 to 0.248	-0.013**	-0.016, -0.010	-0.008**	-0.011, -0.006	-0.051**	-0.069, -0.033	NA	NA
Uganda	Crude	Father	0.252**	0.231 to 0.273	NA	NA	-0.012**	-0.015, -0.010	NA	NA	NA	NA
Uganda	Standardized	Father	0.180**	0.162 to 0.199	NA	NA	-0.012**	-0.015, -0.010	NA	NA	NA	NA
Uganda	Standardized (adjusted)	Father	0.219**	0.200 to 0.238	-0.013**	-0.016, -0.010	-0.008**	-0.011, -0.006	-0.051**	-0.069, -0.033	NA	NA
Uganda	Crude	Both	0.252**	0.232 to 0.273	-0.013**	-0.016, -0.010	-0.009**	-0.012, -0.007	NA	NA	NA	NA
Uganda	Standardized	Both	0.192**	0.175 to 0.210	-0.013**	-0.015, -0.010	-0.009**	-0.011, -0.006	NA	NA	NA	NA
Uganda	Standardized (adjusted)	Both	0.193**	0.175 to 0.210	-0.013**	-0.016, -0.010	-0.008**	-0.011, -0.006	-0.051**	-0.069, -0.033	NA	NA
Uganda	Crude	Mid	0.252**	0.232 to 0.273	NA	NA	NA	NA	NA	NA	-0.022**	-0.026, -0.019

			Constant		Mother's height		Father's height		Household wealth		Mid-parental height	
Country	Estimate	Parent	Constant	95% CI	Coefficient	95% CI	Coefficient	95% CI	Coefficient	95% CI	Coefficient	95% CI
Uganda	Standardized	Mid	0.185**	0.168 to 0.202	NA	NA	NA	NA	NA	NA	-0.021**	-0.025, -0.018
Uganda	Standardized (adjusted)	Mid	0.184**	0.167 to 0.202	NA	NA	NA	NA	-0.050**	-0.068, -0.032	-0.021**	-0.025, -0.018
Zimbabwe	Crude	Mother	0.232**	0.214 to 0.251	-0.013**	-0.016, -0.010	NA	NA	NA	NA	NA	NA
Zimbabwe	Standardized	Mother	0.235**	0.213 to 0.256	-0.012**	-0.016, -0.008	NA	NA	NA	NA	NA	NA
Zimbabwe	Standardized (adjusted)	Mother	0.236**	0.214 to 0.258	-0.010**	-0.015, -0.006	-0.005**	-0.009, -0.002	-0.040**	-0.060, -0.019	NA	NA
Zimbabwe	Crude	Father	0.232**	0.213 to 0.251	NA	NA	-0.008**	-0.011, -0.005	NA	NA	NA	NA
Zimbabwe	Standardized	Father	0.219**	0.195 to 0.242	NA	NA	-0.008**	-0.012, -0.004	NA	NA	NA	NA
Zimbabwe	Standardized (adjusted)	Father	0.231**	0.207 to 0.254	-0.010**	-0.015, -0.006	-0.005**	-0.009, -0.002	-0.040**	-0.060, -0.019	NA	NA
Zimbabwe	Crude	Both	0.232**	0.214 to 0.250	-0.012**	-0.015, -0.009	-0.006**	-0.009, -0.004	NA	NA	NA	NA
Zimbabwe	Standardized	Both	0.223**	0.199 to 0.246	-0.011**	-0.015, -0.007	-0.006**	-0.009, -0.002	NA	NA	NA	NA
Zimbabwe	Standardized (adjusted)	Both	0.224**	0.200 to 0.248	-0.010**	-0.015, -0.006	-0.005**	-0.009, -0.002	-0.040**	-0.060, -0.019	NA	NA
Zimbabwe	Crude	Mid	0.232**	0.214 to 0.250	NA	NA	NA	NA	NA	NA	-0.018**	-0.022, -0.014
Zimbabwe	Standardized	Mid	0.217**	0.194 to 0.240	NA	NA	NA	NA	NA	NA	-0.017**	-0.024, -0.011
Zimbabwe	Standardized (adjusted)	Mid	0.219**	0.196 to 0.242	NA	NA	NA	NA	-0.040**	-0.060, -0.019	-0.016**	-0.022, -0.010

CI, confidence interval; NA, not applicable.

Notes: *p < .1; ** p < .05; *** p < .01. 2 sided. Each row shows parameters from an ordinary least squares model with a binary indicator for stunting as an outcome. The constant from each model shows the proportion stunted. All estimates were weighted using sampling weights which sum up to one for each survey. Standardized estimates were further re-weighted to sum up to the probability-density within each stratum of parental height in the reference population. Parental height was centered around mean parental height in the reference population for Standardized models, and their sample means in Crude models. Household wealth was centered around its sample mean. Standard errors were adjusted for clustering at the PSU-level for Crude estimates, and PSU crossed with parental height for Standardized estimates.

eTable 9. Average maternal height (cm) from Figure 1

	Mean [SD]	95% CI	Observations
All	157.35 [6.8]	(157.29 to 157.4)	575,767
Albania	159.86 [6.5]	(159.41 to 160.3)	2,475
Armenia	159.69 [5.6]	(159.30 to 160.1)	1,576
Azerbaijan	158.63 [5.3]	(158.24 to 159.0)	2,000
Bangladesh	151.10 [5.6]	(150.91 to 151.3)	7,009
Benin	158.79 [6.0]	(158.55 to 159.0)	6,298
Bolivia	151.46 [5.6]	(151.20 to 151.7)	7,734
Brazil	156.10 [6.5]	(155.78 to 156.4)	4,048
Burkina Faso	161.73 [5.8]	(161.50 to 162.0)	6,630
Burundi	155.37 [5.9]	(155.13 to 155.6)	6,040
Cambodia	153.04 [5.2]	(152.79 to 153.3)	4,361
Cameroon	160.44 [6.2]	(160.14 to 160.7)	5,094
Central African Republic	158.83 [6.7]	(158.41 to 159.2)	2,369
Chad	162.75 [6.2]	(162.47 to 163.0)	10,127
Colombia	155.95 [6.0]	(155.79 to 156.1)	15,412
Comoros	156.87 [6.1]	(156.42 to 157.3)	2,519
Congo	158.74 [6.2]	(158.35 to 159.1)	4,472
Congo Democratic Republic	156.90 [6.9]	(156.46 to 157.3)	8,114
Cote D'Ivoire	158.78 [6.1]	(158.34 to 159.2)	3,185
Dominican Republic	159.07 [6.2]	(158.69 to 159.4)	3,205
Egypt	159.37 [5.5]	(159.18 to 159.6)	14,542
Ethiopia	157.23 [6.1]	(156.97 to 157.5)	8,840
Gabon	159.71 [6.4]	(159.16 to 160.3)	3,392
Gambia	162.52 [6.5]	(162.04 to 163.0)	3,145
Ghana	159.07 [5.9]	(158.73 to 159.4)	2,727
Guatemala	148.54 [6.0]	(148.28 to 148.8)	10,908
Guinea	159.37 [6.2]	(159.05 to 159.7)	3,455
Guyana	156.15 [7.5]	(155.24 to 157.1)	1,620
Haiti	159.45 [6.2]	(159.14 to 159.8)	3,752
Honduras	152.90 [6.1]	(152.68 to 153.1)	9,974
India	151.73 [5.9]	(151.67 to 151.8)	231,829
Jordan	158.63 [5.7]	(158.29 to 159.0)	6,297

	Mean [SD]	95% CI	Observations
Kazakhstan	159.26 [5.9]	(158.60 to 159.9)	578
Kenya	159.83 [6.2]	(159.58 to 160.1)	9,032
Kyrgyz Republic	159.33 [5.5]	(159.00 to 159.7)	4,043
Lesotho	157.27 [5.8]	(156.83 to 157.7)	1,330
Liberia	157.10 [6.2]	(156.75 to 157.4)	3,196
Madagascar	153.02 [5.8]	(152.70 to 153.3)	5,220
Malawi	156.19 [5.7]	(155.95 to 156.4)	5,140
Maldives	152.51 [5.5]	(152.13 to 152.9)	2,357
Mali	162.00 [5.9]	(161.67 to 162.3)	4,347
Moldova	161.26 [5.9]	(160.86 to 161.7)	1,343
Morocco	158.54 [5.7]	(158.33 to 158.8)	5,560
Mozambique	154.91 [5.9]	(154.64 to 155.2)	9,484
Myanmar	152.46 [5.3]	(152.19 to 152.7)	4,202
Namibia	161.42 [6.3]	(161.04 to 161.8)	1,794
Nepal	151.27 [5.4]	(150.94 to 151.6)	2,366
Nicaragua	153.50 [6.0]	(153.22 to 153.8)	5,927
Niger	160.55 [5.9]	(160.21 to 160.9)	4,872
Nigeria	158.47 [5.9]	(158.28 to 158.7)	11,298
Pakistan	154.63 [5.8]	(154.25 to 155.0)	4,127
Peru	151.65 [5.5]	(151.44 to 151.9)	9,208
Rwanda	157.04 [6.2]	(156.76 to 157.3)	3,559
Sao Tome & Principe	159.60 [6.9]	(159.01 to 160.2)	1,469
Senegal	163.61 [6.3]	(163.27 to 164.0)	3,738
Sierra Leone	157.53 [6.3]	(157.19 to 157.9)	4,266
South Africa	158.36 [6.0]	(157.85 to 158.9)	1,053
Swaziland	158.81 [5.8]	(158.46 to 159.2)	2,047
Tajikistan	157.79 [5.5]	(157.56 to 158.0)	5,871
Tanzania	157.06 [6.0]	(156.77 to 157.3)	8,984
Timor-Leste	151.69 [5.5]	(151.44 to 151.9)	5,894
Togo	158.94 [6.0]	(158.63 to 159.3)	3,202
Turkey	157.99 [5.6]	(157.57 to 158.4)	1,334
Uganda	158.93 [6.2]	(158.61 to 159.3)	4,413
Uzbekistan	159.51 [6.1]	(158.96 to 160.1)	1,022
Yemen	153.83 [5.9]	(153.63 to 154.0)	13,837

	Mean [SD]	95% CI	Observations
Zambia	157.63 [6.1]	(157.40 to 157.9)	11,570
Zimbabwe	160.38 [6.1]	(160.09 to 160.7)	4,935

CI, confidence interval; SD, standard deviation.

Notes: All estimates were weighted using sampling weights. Pooled estimates were weighted using sampling weights scaled to sum up to one for each survey. Standard errors were adjusted for clustering at the PSU-level.

eTable 10. CPS and maternal height-SPS estimates, ranks, and change in rank from Figure 2

	Estimates				Rank		
	CPS	CPS (95% CI)	SPS	SPS (95% CI)	CPS	SPS	Change
**All**	27.8	(27.5 to 28.1)	23.3	(23.0 to 23.6)			
Guatemala	45.2	(43.7 to 46.9)	14.1	(12.6 to 15.8)	64	17	47
Bangladesh	36.0	(34.3 to 37.7)	21.6	(17.2 to 26.9)	52	28	24
Nepal	32.4	(30.1 to 34.9)	17.4	(12.9 to 22.9)	45	23	22
Bolivia	27.1	(25.4 to 28.8)	12.4	(10.4 to 14.7)	31	12	19
Myanmar	29.1	(27.2 to 31.2)	16.8	(12.7 to 21.9)	38	21	17
India	37.9	(37.6 to 38.3)	25.2	(24.5 to 26.0)	55	38	17
Pakistan	33.5	(30.7 to 36.5)	23.5	(20.6 to 26.6)	49	32	17
Cambodia	33.1	(31.2 to 35.1)	23.9	(18.9 to 29.8)	47	33	14
Peru	17.9	(16.7 to 19.3)	5.8	(5.0 to 6.7)	14	1	13
Honduras	22.4	(21.3 to 23.6)	9.8	(8.9 to 10.8)	20	8	12
Nicaragua	24.5	(22.8 to 26.3)	13.7	(12.0 to 15.5)	26	15	11
Mozambique	43.4	(41.7 to 45.0)	34.0	(32.2 to 35.9)	62	55	7
Yemen	45.9	(44.3 to 47.6)	36.0	(33.3 to 38.8)	65	59	6
Timor-Leste	43.7	(41.8 to 45.6)	35.1	(30.4 to 40.1)	63	57	6
Tanzania	30.4	(28.8 to 31.9)	25.0	(23.7 to 26.3)	40	36	4
Malawi	31.9	(30.3 to 33.6)	25.3	(23.2 to 27.4)	43	39	4
Brazil	13.4	(12.1 to 14.8)	8.2	(7.4 to 9.2)	9	6	3
Colombia	12.5	(11.8 to 13.3)	8.2	(7.6 to 8.8)	7	4	3
Maldives	13.1	(11.5 to 14.9)	8.2	(5.5 to 12.0)	8	5	3
Rwanda	38.2	(36.3 to 40.1)	33.0	(30.6 to 35.5)	56	54	2
Burundi	51.0	(49.3 to 52.7)	39.9	(37.8 to 42.1)	67	65	2
Madagascar	48.8	(46.6 to 51.0)	39.2	(36.1 to 42.4)	66	64	2
Zambia	40.0	(38.8 to 41.3)	35.8	(34.5 to 37.1)	59	58	1
Guyana	20.8	(18.4 to 23.5)	13.8	(11.7 to 16.3)	17	16	1
Nigeria	33.2	(31.7 to 34.7)	29.0	(27.9 to 30.2)	48	48	0
Jordan	8.1	(6.9 to 9.4)	7.0	(6.0 to 8.2)	3	3	0
Dominican Republic	7.1	(6.1 to 8.4)	6.4	(5.4 to 7.6)	1	2	-1
Liberia	30.1	(27.9 to 32.3)	25.7	(23.4 to 28.1)	39	40	-1
Ethiopia	35.9	(34.0 to 37.8)	32.0	(30.2 to 33.8)	51	52	-1
Congo Democratic Republic	41.8	(40.0 to 43.7)	36.4	(34.7 to 38.2)	60	61	-1
Kazakhstan	13.9	(10.7 to 17.7)	12.2	(9.4 to 15.6)	10	11	-1
Uganda	25.0	(23.5 to 26.7)	22.5	(21.1 to 24.0)	28	29	-1
Sierra Leone	37.7	(35.6 to 39.7)	34.6	(32.6 to 36.6)	54	56	-2
Lesotho	32.3	(29.2 to 35.6)	27.0	(23.8 to 30.4)	44	46	-2
Congo	23.5	(21.7 to 25.4)	20.3	(18.7 to 22.1)	23	25	-2
Tajikistan	15.5	(14.3 to 16.8)	13.6	(12.4 to 14.9)	11	14	-3
South Africa	22.6	(19.3 to 26.4)	19.9	(16.6 to 23.6)	21	24	-3
Ghana	18.5	(16.4 to 20.7)	16.8	(15.0 to 18.7)	16	20	-4
Swaziland	27.7	(25.6 to 29.9)	25.0	(22.9 to 27.2)	33	37	-4
Central African Republic	40.0	(37.8 to 42.3)	37.1	(35.0 to 39.2)	58	63	-5
Turkey	10.6	(8.7 to 13.0)	10.1	(7.9 to 12.8)	5	10	-5
Kenya	25.5	(24.2 to 26.8)	24.4	(23.2 to 25.7)	29	34	-5
Albania	10.2	(8.6 to 12.1)	9.9	(8.3 to 11.9)	4	9	-5
Togo	26.8	(24.8 to 28.9)	24.7	(22.9 to 26.6)	30	35	-5
Morocco	23.2	(21.6 to 25.0)	21.3	(19.9 to 22.7)	22	27	-5
Armenia	8.0	(6.4 to 9.9)	8.5	(6.9 to 10.4)	2	7	-5
Gabon	17.2	(14.9 to 19.8)	16.0	(14.4 to 17.8)	12	18	-6
Cote D'Ivoire	28.6	(26.5 to 30.7)	26.2	(24.3 to 28.3)	36	42	-6
Haiti	17.7	(16.1 to 19.6)	16.5	(15.1 to 17.9)	13	19	-6
Niger	41.9	(39.9 to 43.8)	41.9	(40.1 to 43.7)	61	67	-6
Cameroon	32.5	(30.5 to 34.5)	32.1	(30.5 to 33.7)	46	53	-7

	Estimates				Rank		
	CPS	CPS (95% CI)	SPS	SPS (95% CI)	CPS	SPS	Change
Uzbekistan	37.3	(33.6 to 41.1)	36.3	(32.6 to 40.1)	53	60	-7
Egypt	22.3	(21.0 to 23.6)	21.2	(20.3 to 22.2)	19	26	-7
Kyrgyz Republic	18.3	(16.4 to 20.3)	17.1	(15.6 to 18.7)	15	22	-7
Zimbabwe	23.6	(22.1 to 25.1)	23.4	(21.9 to 25.0)	24	31	-7
Sao Tome & Principe	31.3	(28.1 to 34.6)	30.3	(27.2 to 33.6)	42	49	-7
Moldova	11.8	(10.1 to 13.9)	12.4	(10.5 to 14.7)	6	13	-7
Benin	29.1	(27.6 to 30.7)	26.7	(25.4 to 28.0)	37	44	-7
Chad	39.5	(37.9 to 41.2)	41.3	(40.0 to 42.6)	57	66	-9
Comoros	30.7	(28.2 to 33.3)	30.8	(27.6 to 34.2)	41	50	-9
Azerbaijan	27.4	(23.9 to 31.2)	26.5	(23.4 to 29.7)	32	43	-11
Guinea	27.8	(26.0 to 29.6)	26.9	(25.1 to 28.7)	34	45	-11
Namibia	21.3	(18.9 to 23.9)	22.5	(20.3 to 24.8)	18	30	-12
Burkina Faso	35.0	(33.5 to 36.6)	36.4	(35.0 to 37.9)	50	62	-12
Senegal	28.0	(25.8 to 30.2)	31.6	(29.5 to 33.8)	35	51	-16
Mali	24.4	(22.7 to 26.1)	25.7	(24.0 to 27.5)	25	41	-16
Gambia	24.9	(22.7 to 27.2)	27.1	(25.0 to 29.2)	27	47	-20

CI, confidence interval; CPS, crude prevalence of stunting; SPS, standardized prevalence of stunting.

Notes: All estimates were weighted using sampling weights. Pooled estimates were weighted using sampling weights scaled to sum up to one for each survey. Standard errors were adjusted for clustering at the PSU-level.

eTable 11. Spearman's rank correlation coefficients of CPS and SPS and other aggregate measures of child health from Figure 3

	ALRI	Diarrhea	Anemia	U5MR	CMR	DPT3
CPS	0.59	0.62	0.59	0.61	0.60	-0.45
	(n=60)	(n=60)	(n=65)	(n=65)	(n=65)	(n=65)
Maternal height-SPS	0.79	0.80	0.72	0.80	0.79	-0.49
	(n=60)	(n=60)	(n=65)	(n=65)	(n=65)	(n=65)
Adjusted maternal height-SPS	0.78	0.80	0.72	0.79	0.78	-0.51
	(n=60)	(n=60)	(n=65)	(n=65)	(n=65)	(n=65)

CPS, crude prevalence of stunting; SPS, standardized prevalence of stunting.

Notes: Acute lower respiratory infections death rate for children under five (ALRI); Diarrhea death rate for children under five (Diarrhea); Prevalence of anemia among children under five (Anemia); Under-five mortality rate (U5MR); Child mortality rate (CMR); Diphtheria-pertussis-tetanus vaccination coverage for children 1 – 2 years old (DPT3). n refers to number of countries with available data. Adjusted maternal height-SPS adjusts for current living standards.
